# Vapor‐Phase Detection of Perfluoroalkyl Acids using Amplifying Fluorescent Polymers

**DOI:** 10.1002/smtd.70812

**Published:** 2026-07-21

**Authors:** Jesús Castro‐Esteban, Ulysse J. Gross, Timothy M. Swager

**Affiliations:** ^1^ Department of Chemistry Massachusetts Institute of Technology Cambridge Massachusetts USA

**Keywords:** conjugated polymers, fluorescence, perfluoroalkyl acids, vapor‐phase

## Abstract

Perfluoroalkyl acids are a subclass of persistent toxic per‐ and polyfluoroalkyl substances (PFAS) whose volatility enables airborne exposure pathways that remain largely unmonitored. Therefore, suitable technologies are urgently needed for real‐time monitoring of PFAS acids in the vapor phase. Herein, we report a new platform for selective vapor‐phase detection of volatile perfluoroalkyl acids using amplifying fluorescent polymers. The designs incorporate highly fluorinated moieties to create a fluorous microenvironment that enhances selectivity and pyridine‐based selectors that react with volatile PFAS acids through proton‐transfer reactions. Ratiometric fluorescence detection was employed in sensing experiments, and polymer thin films exhibited rapid responses (<5 s) to PFAS acids vapor exposure, with the induced signal persisting for more than 15 min. The polymer **C_8_/C_4_PF‐Py** emerged as the most sensitive material, exhibiting vapor‐phase detection limits of 47.8 ppm, 2.38 ppm, and 450 ppb for TFA, PFBA, and GenX, respectively. This work establishes a versatile, rapid, and selective platform for vapor‐phase detection of PFAS acids. The materials can be coated on a variety of surfaces, and their fluorescence changes can be easily monitored, thereby reducing expensive instrumentation dependence and laboratory testing, providing a practical pathway toward routine monitoring and regulation of airborne PFAS acids.

## Graphical abstract

Amplifying fluorescent fluorous polymers respond to PFAS vapors in under 5 s. The fluorophilic nature of the polymers results in strong binding and selectivity.

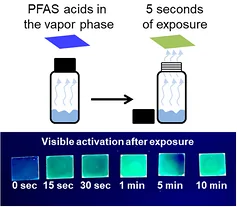

## 1 Introduction

**FIGURE 1:**
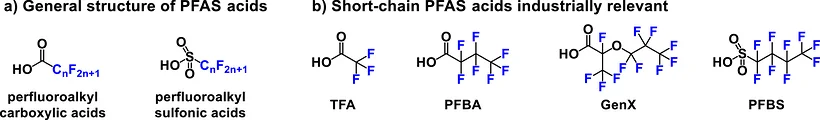
(a) General structure of PFAS acids. (b) PFAS acids industrially relevant used in this work.

Per‐ and polyfluoroalkyl substances (PFAS) comprise a large group of synthetic fluorinated organic compounds characterized by exceptionally strong carbon‐fluorine bonds, which impart them exceptional chemical and thermal stability [1, 2, 3, 4, 5]. Within this group, perfluoroalkyl acids are distinguished by highly fluorinated tails and polar acidic headgroups that confer water solubility (Figure 1a). This amphiphilic structure, combined with their extreme resistance to degradation, pronounced bioaccumulation, and high environmental mobility, renders PFAS acids a persistent threat to ecosystems and public health [6, 7, 8, 9].

PFAS acids can exhibit toxicity even at trace concentrations, and their persistence and widespread distribution have raised global concern, as human exposure occurs predominantly through food and drinking water [10, 11, 12, 13]. Indeed, biomonitoring studies consistently report detectable levels of perfluoroalkyl acids in the blood of the general population [14, 15]. Consequently, in response to increasing concern over PFAS acids contamination, significant progress has been made in their detection and remediation in aqueous environments. Sensitive detection in water has been achieved through validated analytical methods [16, 17] as well as emerging electrochemical and optical sensors [18, 19, 20, 21, 22, 23], while complementary strategies for capture [24, 25, 26, 27, 28, 29] and degradation [30, 31, 32, 33, 34, 35, 36] have also been extensively investigated.

Despite the toxicity and bioaccumulation concerns, PFAS acids remain indispensable in several industrial applications, including fluoropolymer production and semiconductor manufacturing [37, 38, 39, 40]. Following the phase‐out of perfluorooctanoic acid (PFOA) and perfluorooctane sulfonic acid (PFOS) in the early 2000s, these highly toxic compounds were largely replaced by shorter‐chain perfluoroalkyl acids (Figure 1b), including perfluorobutanoic acid (PFBA), perfluorobutane sulfonic acid (PFBS), and hexafluoropropylene oxide dimer acid (GenX) [41]. These replacements were introduced under the assumption of reduced toxicity and shorter biological half‐lives. However, increasing evidence suggests that their higher volatility may facilitate airborne exposure via gradual evaporation from products and surfaces, thereby enhancing environmental dispersion and human exposure pathways [42, 43, 44, 45, 46, 47].

Although PFAS acids in drinking water are regulated, airborne exposure remains largely unregulated, primarily due to the complexity and limitations of existing detection methods. The U.S. Environmental Protection Agency has proposed two analytical approaches for airborne PFAS acids monitoring, including OTM‐45 for stationary‐source monitoring based on isotope‐dilution LC‐MS/MS and thermal desorption GC‐MS/MS [48]. These methods offer high sensitivity and selectivity for volatile PFAS, however, they require costly instrumentation, are time‐consuming, and involve complex sampling protocols. To date, there are no alternative methods for the selective vapor‐phase detection of PFAS acids, despite the implementation of optical sensing strategies for other volatile acid analytes, including acetic acid, formic acid, and trifluoroacetic acid [49, 50, 51, 52, 53, 54].

Our group has previously demonstrated the use of amplifying fluorescent polymer (AFP) platforms for trace‐level vapor‐phase detection of ketones and explosives, with successful implementation in commercial sensing devices [55, 56, 57, 58, 59]. Additionally, AFPs have demonstrated selective detection of PFAS acids in water at ultratrace concentrations (80 ppt) [60].

Herein, we report an AFP platform for selective and sensitive vapor‐phase detection of volatile perfluoroalkyl acids, where analyte uptake occurs at gas‐solid interfaces through adsorption and partitioning into fluorinated regions, rather than being governed by diffusion, hydration effects, and polymer free volume as in aqueous systems. These distinct environments impose different design requirements, and the materials reported have structures containing fluorous fluorene, indenofluorene, and indenoindene monomers, which create a fluorophilic microenvironment that enhances selectivity for PFAS acids. The polymers contain pyridine‐based selector groups (thienylpyridine and pyridine units) that react with volatile PFAS acids through proton‐transfer reactions, which introduce a strong charge‐transfer state in the polymers that capture mobile excitons to provide an amplified response. Five AFPs were synthesized (PF‐Py*, **PF‐Py**, **PIF‐PF‐Py**, **PII‐Py**, and **C_8_/C_4_PF‐Py**) and their sensing responses were evaluated to volatile PFAS acids (TFA, GenX, PFBA, and PFBS) as well as non‐fluorous acid compounds such as acetic acid, butyric acid, octanoic acid, and formic acid.

Ratiometric fluorescence detection was employed in the sensing experiments, wherein we monitor both the unactivated polymer emission maxima and the activated red‐shifted emission maxima upon PFAS acids reaction. The lower energy of the activated charge transfer states create exciton trapping sites, and only a small fraction of the pyridines need to be protonated to dominate the signal. Polymer thin films exhibit rapid responses to PFAS acids vapors with 5 s exposures, and the signals persist for more than 15 min. In contrast, exposure to non‐fluorous acid compounds did not produce measurable responses, demonstrating the high selectivity of the sensing platform. Among the polymers investigated, **C_8_/C_4_PF‐Py** emerged as the most sensitive material, exhibiting limits of detection of 47.8 ppm (TFA), 2.38 ppm (PFBA), and 450 ppb (GenX) within 5 s of vapor exposure and 69 ppm (PFBS) within 5 min of exposure. Collectively, this work establishes a versatile, rapid, and selective platform for vapor‐phase detection of PFAS acids. The fluorinated AFPs can be coated on a variety of surfaces, and their fluorescence changes can be easily monitored, thereby reducing expensive instrumentation dependence and laboratory testing, providing a practical pathway toward routine monitoring and future regulation of airborne PFAS acids.

## 2 Results and Discussion

### 2.1 Monomer Designs

In contrast to aqueous‐phase AFPs sensing, where the response is strongly influenced by PFAS diffusion through solvated media and polymer free volume [60], vapor‐phase PFAS sensing is dominated by interfacial adsorption and partitioning into fluorinated regions. Therefore, to promote effective vapor‐phase PFAS uptake, the polymer designs prioritize high fluorine content to enhance fluorophilic interactions with the analytes, incorporating perfluorooctyl chain substituents. In addition, shorter chains (perfluorobutyl groups) were also incorporated to enhance the interactions with short‐chain PFAS acids, which are generally more challenging to detect.

**FIGURE 2:**
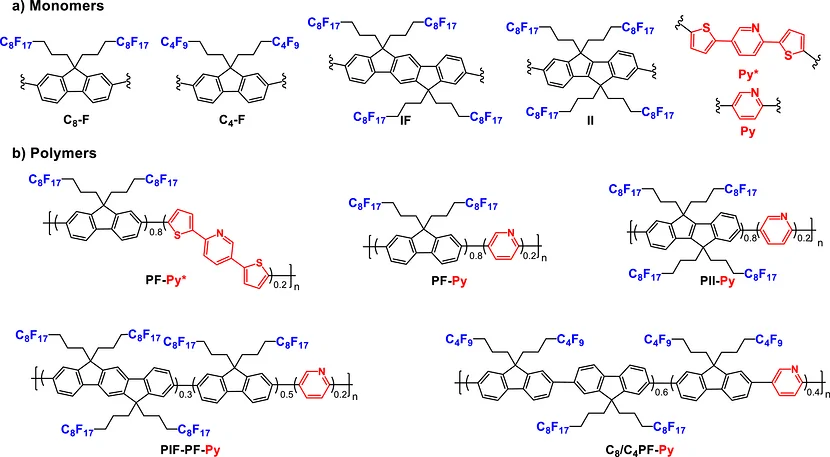
(a) Highly fluorinated monomers as well as pyridine‐based selectors, and (b) sensing polymers used in this work.

Fluorene, 6,12‐Dihydroindeno[1,2‐*b*]fluorene, and 5,10‐Dihydroindeno[2,1‐*a*]indene were selected as monomers due to their shared five‐membered ring core, which can be used to create a highly conjugated polymer backbone with proven effectiveness for the design of emissive materials. Different types of these polycyclic aromatic scaffolds can be easily synthesized through alkylation reactions, thereby affording a method for incorporating perfluoroalkyl chains to tune the fluorine content. Monomers bearing perfluorooctylpropyl (**C_8_‐F**, **IF**, and **II**) and perfluorobutylpropyl (**C_4_‐F**) chains were synthesized, along with two pyridine‐based selectors: thienylpyridine (Py*) and pyridine (**Py**) (Figure 2a). Detailed synthetic procedures are provided in the Supporting Information.

Polymer Synthesis. PF‐Py*, **PF‐Py**, **PIF‐PF‐Py**, **PII‐Py**, and **C_8_/C_4_PF‐Py** were synthesized through palladium‐catalyzed Suzuki cross‐coupling polycondensation reactions (Figure 2b). PF‐Py* and **PF‐Py** were previously reported [60], and differ only in the pyridine‐based selector, thereby enabling a direct comparison of their sensing responses. Polymer **C_8_/C_4_PF‐Py** incorporates two fluorine monomers with different perfluoroalkyl chain lengths, allowing assessment of how side‐chain structure influences sensing performance. The **PIF‐PF‐Py** composition combines indenofluorene and fluorene monomers, whereas **PII‐Py** consists exclusively of indenoindene monomers. These latter materials were targeted to evaluate the rigidity of the polymer backbone and different fluorine content in the materials during sensing.

The high fluorous character of the monomers limits their solubility in non‐fluorous solvents. Therefore, the Suzuki polymerizations were carried out at 90°C for 72 h in PhCF_3_, to ensure solubility of the growing polymer chains. The polymers were purified by precipitation in methanol, with two subsequent redissolution‐reprecipitation cycles (PhCF_3_/methanol), and a final precipitation in acetone. For more synthetic details, see the Supporting Information. The relative molecular weight (*M_n_*) and polydispersity (*Đ*) of the polymers were determined by gel permeation chromatography (GPC) in THF (Table S1 and Figure S2). PF‐Py*, **PF‐Py**, **PIF‐PF‐Py**, and **C_8_/C_4_PF‐Py** remained sufficiently soluble in THF for this analysis despite their high fluorine contents (56.4, 58.5, 59.9, and 53.3 wt.% F, respectively). In contrast, **PII‐Py** with the highest fluorine content (62.6 F wt.%) has limited solubility in common organic solvents (CHCl_3_, THF) and PhCF_3_. Consequently, only the THF‐soluble fraction of this material was used in subsequent studies.

### 2.2 Optical Properties

UV–vis absorption and fluorescence spectra were collected for the five polymers in THF solutions and spin‐cast thin films (Figure S3). Relevant photophysical parameters, including absorption and emission maxima, are summarized in Table S2. PF‐Py* and **PF‐Py** were originally studied in PhCF_3_ solutions [60], and they displayed comparable optical features in THF, and their behavior in solid state matches our previous studies. **C_8_/C_4_PF‐Py** exhibited very similar absorption and emission profiles in both solution and thin films, which suggests that the mixed lengths of the perfluoroalkyl chains suppress ordering in the solid state. AFPs **PIF‐PF‐Py** and **PII‐Py** displayed red‐shifted absorption and emission relative to polyfluorene‐based polymers. This behavior is a result of extended π‐conjugation enforced by the framework, which reduces the optical band gap and shifts the electronic transitions to lower energies.

### 2.3 Target Analytes

Trifluoroacetic acid (TFA), perfluorobutanoic acid (PFBA), hexafluoropropylene oxide dimer acid (GenX), and perfluorobutane sulfonic acid (PFBS) were selected for the sensing studies as PFAS acids of interest. These short‐chain perfluoroalkyl acids are widely used in industrial applications as surfactants, in fluoropolymer production, and in semiconductor photolithography and etching processes [37, 38, 39, 40]. TFA, the shortest and highly volatile perfluoroalkyl acid, has also emerged as a potential environmental and public health concern and was therefore included in this study [61]. PFBA and GenX also exhibit high vapor pressures, whereas PFBS displays moderate to low volatility but remains relevant for monitoring because of its widespread use. The selectivity of our methodology to PFAS acids was evaluated by experimenting with acetic acid (AcOH), butyric acid (BA), octanoic acid (OA), and formic acid (FA), which exhibit comparable vapor pressures. Vapor pressure data and calculated airborne concentrations are summarized in Table S3.

### 2.4 Sensing Experiments

**FIGURE 3:**
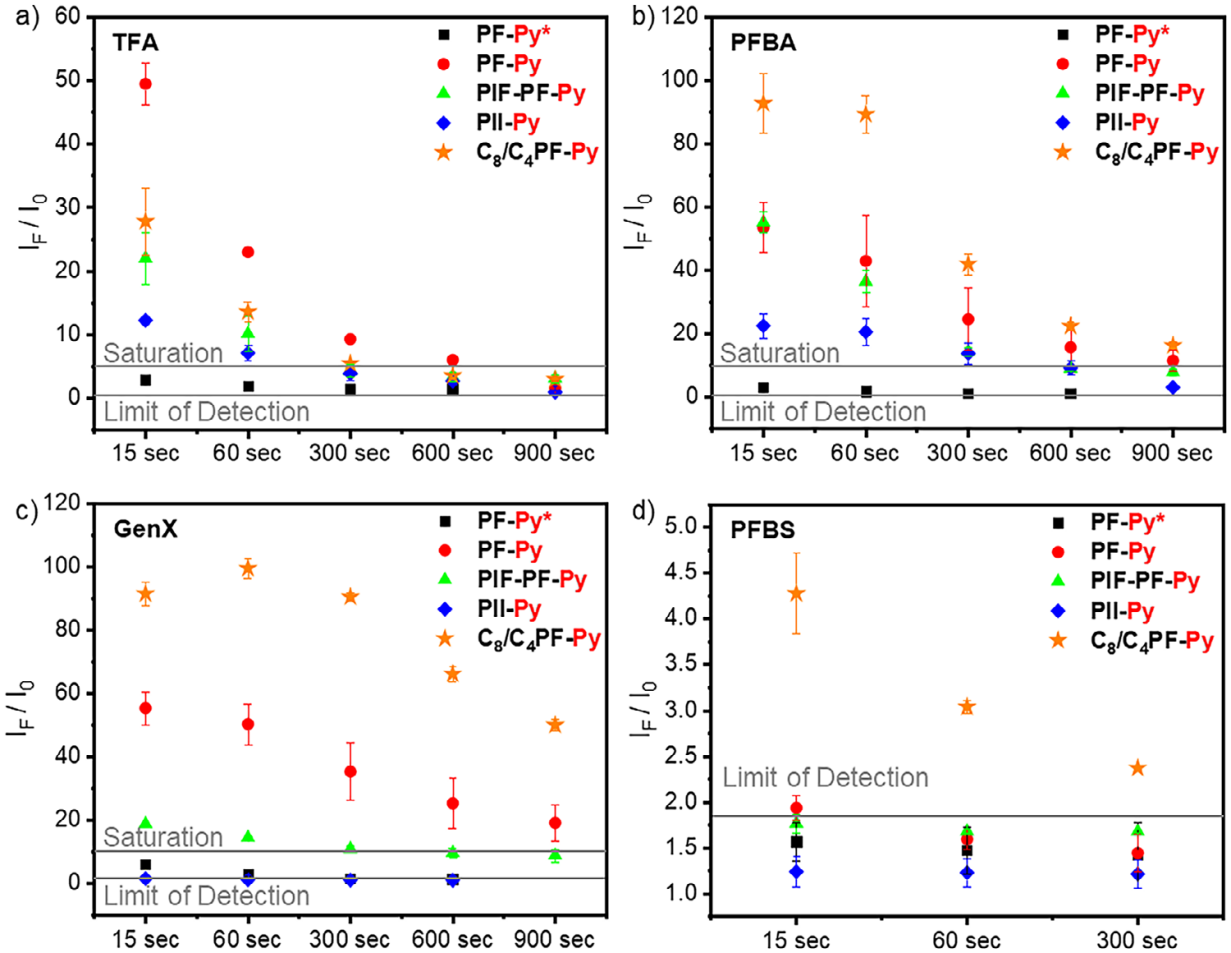
Sensing responses to: (a) TFA, (b) PFBA, (c) GenX, and (d) PFBS using PF‐Py*, **PF‐Py**, **PIF‐PF‐Py**, **PII‐Py**, and **C_8_/C_4_PF‐Py**. Plots showing the difference of the final and initial ratiometric fluorescence signal as a function of time after PFAS acid exposure (see text). Wavelenghts monitored for PF‐Py* (488 and 564 nm), **PF‐Py** (428 and 498 nm), **PIF‐PF‐Py** (430 and 540 nm), **PII‐Py** (462 and 528 nm), and **C_8_/C_4_PF‐Py** (419 and 487 nm).

**FIGURE 4:**
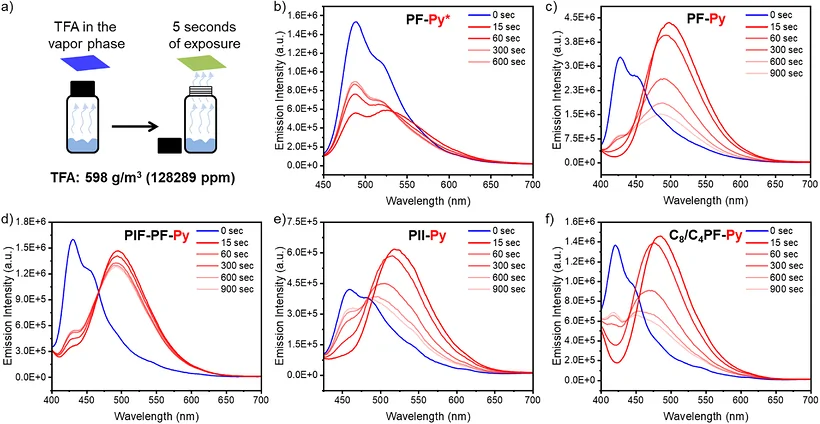
(a) Graphical abstract of polymer thin films exposure to PFAS acids in the vapor phase. The unactivated polymer emission is shown in blue. Red spectra represent fluorescence recovery over time, with each trace corresponding to a different post‐exposure time (increasing transparency indicates longer times). Results after a 5 s exposure to TFA vapor using: (b) PF‐Py*, (c) **PF‐Py**, (d) **PIF‐PF‐Py**, (e) **PII‐Py**, and (f) **C_8_/C_4_PF‐Py**.

AFP sensing studies for TFA, PFBA, GenX, and PFBS were conducted using polymer thin films, and recording fluorescence spectra before and after exposure using a bifurcated fiber optic accessory. Ratiometric fluorescence detection was employed, monitoring both intensity at the unactivated polymer emission maxima and the red‐shifted emission maxima originating from the acid‐activated selector sites. Fluorescence was analyzed immediately after exposing thin films to PFAS acid vapor, recording the emission at defined time intervals (15, 60, 300, 600, and 900 s). The sensing responses of PF‐Py*, **PF‐Py**, **PIF‐PF‐Py**, **PII‐Py**, and **C_8_/C_4_PF‐Py** are summarized in Figure 3, with plots of the ratiometric signal intensities of the final to initial fluorescence as a function of time. All experiments were performed in triplicate using three independent polymer thin films for each compound. Controlled headspace conditions were established by placing 1 mL of analyte in a 4 mL vial, which was then enclosed within a sealed 20 mL vial. After vapor pressure equilibration for 15 min, the vial was opened, and polymer films were exposed to the vapor for 5 s. Figure 4a illustrates the procedure followed during the experiments.

Initial screening was performed by exposing polymer thin films to TFA vapor at a headspace concentration of 598 g/m^3^ (128289 ppm) for 5 s (Figure 2a). PF‐Py* films exhibited a transient visual fluorescence change from green to orange, followed by very rapid recovery of the initial emission. As shown in Figure 4b, only minimal activation was detected 15 s after the sample had been exposed to the vapor, with near‐complete recovery of the initial signal within 60 s. In contrast, the other polymers exhibited saturation of the red‐shifted emission within 15 s, followed by slow, gradual recovery of the initial fluorescence over approximately 15 min, with most films remaining partially activated (Figure 4c–f). The gradual fluorescence recovery is attributed to the high volatility of TFA, which eventually desorbs from the polymer matrix. To further investigate the fluorescence recovery, an additional experiment was performed in which thin films of **C_8_/C_4_PF‐Py** were exposed to TFA vapor for 5 s from a freshly opened commercial bottle (Sigma Aldrich, 100 mL), showing visible activation to the naked eye in the thin films after 30 min (Figure S4).

An additional study was performed by exposing non‐fluorinated polymer (**AlkylPF‐Py**) films to TFA vapor to compare the response with the fluorinated **PF‐Py** and understand the role of hydrophobic vs fluorophilic interactions (Figure S5). **AlkylPF‐Py** films exhibited a strong initial response without signal saturation, with a fast recovery to the initial emission after 5 min, while the fluorinated **PF‐Py** showed signal saturation after the exposure during 5 min with a minimal recovery of the initial emission after 10 min. This demonstrates that fluorophilic interactions play a crucial role during PFAS acids adsorption and partitioning to enhance the sensitivity and response time.

Exposure to PFBA vapor at a concentration of 115 g/m^3^ (13158 ppm) resulted in rapid activation of the sensing response within 5 s of exposure (Figure 3b and Figure S5), followed by signal saturation over a period of 10 min for all polymers, except PF‐Py*, which did not reach complete activation after 60 s. A similar response profile was observed upon 5 s exposure to GenX vapor at 47.9 g/m^3^ (3553 ppm) (Figure 3c and Figure S6). However, **PII‐Py** did not display red‐shifted activation, but instead undergoes a slight fluorescence quenching, suggesting polymer‐GenX interactions that do not involve protonation of this polymer's pyridine moieties but instead promote nonradiative exciton decay in this system. Additionally, **PF‐Py** and **C_8_/C_4_PF‐Py**, with a similar backbone, exhibited near‐identical response kinetics and saturation behavior indicating that the length of the fluorocarbon sidechains has only a very small effect on sensing performance.

PFBS exhibits lower vapor pressure relative to other PFAS acids studied, and longer exposure times (5 min) were required to induce measurable fluorescence responses (Figure 3d and Figure S7). These experiments were conducted using 1 mg of PFBS deposited on a cotton gauze, whose high surface area maintains a headspace that can rapidly reach equilibrium with polymer exposures [58]. Under these conditions, only **PF‐Py** and **C_8_/C_4_PF‐Py** exhibited detectable activation at a concentration of 0.85 g/m^3^ (69 ppm). Notably, only **C_8_/C_4_PF‐Py** retained a red‐shifted emission signal after 5 min postexposure, suggesting that mixed heterogeneous perfluoroalkyl chain lengths promote enhanced PFAS acids transport and trapping. An additional study was performed for the vapor‐phase detection of PFOA using **C_8_/C_4_PF‐Py** (Figure S9). However, no fluorescence response was observed even after 5 min of exposure, which is consistent with the extremely low vapor pressure of PFOA. This negligible response to PFOA vapor, despite being more fluorophilic than PFBA, highlights the dominant role of volatility in vapor‐phase sensing, where lower vapor pressure limits analyte availability, while fluorophilic interactions primarily influence uptake once the PFAS reach the polymer interface. Additionally, the observed non‐monotonic sensitivity as a function of fluorine content in the polymers reflects a trade‐off between increased PFAS affinity and reduced analyte transport within more highly fluorinated films such as **PII‐Py**.

Selectivity was further evaluated using non‐fluorous acid vapors, including acetic acid (AcOH, 36.8 g/m^3^, 15 000 ppm), butyric acid (BA, 2.04 g/m^3^, 566 ppm), octanoic acid (OA, 0.29 g/m^3^, 49.3 ppm), and formic acid (FA, 111 g/m^3^, 58 947 ppm). In all cases, no measurable fluorescence changes were observed after 5 s of exposure, demonstrating the high selectivity of the polymers to PFAS acids even at high concentrations of the non‐fluorescent analogues (Figure S8).

### 2.5 Limit of Detection (LOD)

Vapor‐phase concentrations in the headspace were calculated using Raoult's law in combination with the ideal gas law. PFAS acids solutions were prepared in chloroform, and the partial vapor pressure of each analyte was calculated from its mole fraction in solution and the vapor pressure of the pure compound. Chloroform was selected as the solvent due to negligible interaction with PFAS acids, minimizing non‐ideal gas behavior. Corresponding headspace concentrations were calculated assuming ideal gas behavior. Systematic dilution of the solutions enabled precise control over analyte concentration in the vapor phase. More details about the calculations of the concentrations in the vapor phase are in Section S5.

**FIGURE 5:**
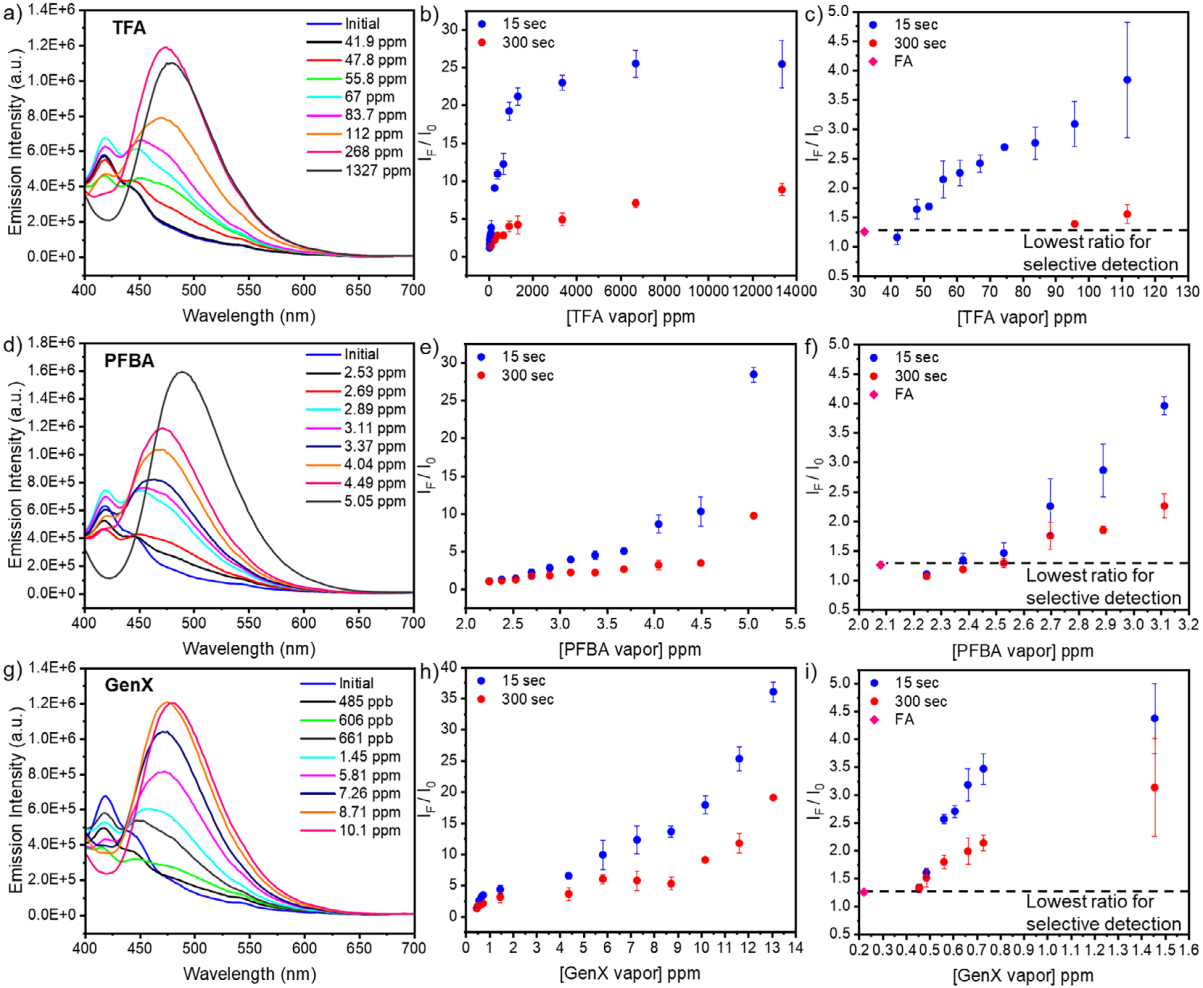
Fluorescence spectra of **C_8_/C_4_PF‐Py** thin films upon 5 s exposure to PFAS acids vapor: (a) TFA, (d) PFBA, and (g) GenX. Calibration curves including the lowest ratio for selective detection: (b) and (c) TFA, (e) and (f) PFBA, and (h) and (i) GenX.

**C_8_/C_4_PF‐Py** emerged as the most effective sensing material, exhibiting rapid and sustained responses to multiple PFAS acids, along with no response to nonfluorous acid compounds. Calibration curves were obtained by plotting the change between the initial and final ratiometric fluorescence responses as a function of the calculated vapor‐phase concentrations in the headspace (Figure 5). Experimental limits of detection (Table 1) were determined from the minimum detectable response at 15 and 300 s after PFAS acids exposure, using the maximum signal observed for formic acid as a conservative threshold. **C_8_/C_4_PF‐Py** displays limits of detection in the vapor phase after 5 s of exposure of 47.8 ppm, 2.38 ppm, and 450 ppb for TFA, PFBA, and GenX, respectively, and after 5 min of exposure of 69 ppm for PFBS. Deviations from linearity were observed in the calibration curves at higher PFAS acids concentrations, particularly for GenX, suggesting complex interactions between the analyte and the polymer chains, including analyte clustering or changes in polymer morphology.

**TABLE 1:** Experimental limits of detection using **C_8_/C_4_PF‐Py** polymer thin films.

Detection time	TFA^a^	PFBA^a^	GenX^a^	PFBS^b^
15 s	47.8 ppm 223 mg/m^3^	2.38 ppm 20.8 mg/m^3^	0.45 ppm 6.14 mg/m^3^	69.0 ppm 0.85 mg/m^3^
300 s	95.7 ppm 446 mg/m^3^	2.53 ppm 22.1 mg/m^3^	0.45 ppm 6.14 mg/m^3^	69.0 ppm 0.85 mg/m^3^

^a^ 5 s of exposure to vapor.

^b^ 5 min of exposure to vapor.

Given the importance of analyte diffusion in vapor‐phase detection, using **C_8_/C_4_PF‐Py** films of varying thickness were examined (Figure S11). The experiment was performed exposing the films during 5 s to 60.9 ppm of TFA vapor. The observed dependence of response on film thickness is consistent with thickness‐dependent PFAS transport within the polymer thin films, where thinner films facilitate more efficient analyte access to the pyridine selectors and result in slightly enhanced fluorescence responses.

## 3 Conclusions

In conclusion, we have developed the first selective platform for vapor‐phase detection of perfluoroalkyl acids using amplifying fluorescent polymers. The highly fluorinated conjugated polymers create fluorous microenvironments that facilitate PFAS uptake at gas‐solid interfaces through adsorption and fluorophilic partitioning. Subsequent analyte diffusion to the pyridine selectors followed by proton‐transfer reactivity generates amplified fluorescence responses through low‐energy emissive trapping sites. The resulting thin films respond within 5 s to multiple industrially relevant PFAS acids, while exhibiting no measurable response towards nonfluorous organic acids, highlighting the intrinsic selectivity of the designs.

Structure‐property relationships reveal that both fluorine content and polymer architecture collectively govern gas‐solid interfacial adsorption, analyte diffusion and the resulting amplified fluorescence response. **PF‐Py** and **C_8_/C_4_PF‐Py** exhibit the strongest and most reproducible responses. **C_8_/C_4_PF‐Py** emerges as the optimal sensing material, which we propose to be attributed to the mixed perfluoroalkyl chain lengths. This latter feature likely promotes enhanced perfluoroalkyl acids trapping and signal persistence within the fluorous polymer matrix. **C_8_/C_4_PF‐Py** displays limits of detection in the vapor phase after 5 s of exposure of 47.8 ppm, 2.38 ppm, and 450 ppb for TFA, PFBA, and GenX, respectively, and after 5 min of exposure of 69 ppm for PFBS.

This work demonstrates a robust method for rapid, sensitive, and selective vapor‐phase detection of perfluoroalkyl acids. To further improve detection limits, sensitivity can be enhanced through optimization of film morphology and thickness to balance PFAS uptake and diffusion within the polymer thin films as well as through engineering of device architectures that promote analyte delivery and preconcentration at the polymer interface. The materials can be coated on a variety of surfaces, and their fluorescence changes can be easily monitored. Therefore, PFAS acids vapor can be detected without reliance on complex instrumentation, and our methods enable on‐site monitoring of airborne contaminants, avoiding the complexity and limitations of existing methods.

## Conflicts of Interest

A provisional patent has been filed.

## Supporting information




**Supporting File**: smtd70812‐sup‐0001‐SuppMat.pdf.

## Data Availability

The data that support the findings of this study are available in the Supporting Information of this article.
