# Supplementary material for: Vapor‐Phase Detection of Perfluoroalkyl Acids using Amplifying Fluorescent Polymers
Source: Small Methods. 2026 Jul 21;10(16):e70812. doi: 10.1002/smtd.70812 (PMC13505700; doi:10.1002/smtd.70812)
Supplement: Supplementary file 1 [file SMTD-10-e70812-s001.pdf]

Supporting Information (SI) for:

Vapor-Phase Detection of Perfluoroalkyl Acids using Amplifying Fluorescence Polymers

Jesús Castro-Esteban, Ulysse J. Gross, and Timothy M. Swager*

Department of Chemistry, Massachusetts Institute of Technology, Cambridge, MA, 02139, USA
*Corresponding Author: tswager@mit.edu

1. **Materials and instruments S2**

2. **Synthesis of monomers and polymers S2**
   - **2.1. Synthesis of monomers S3**
   - **2.2. Synthesis of polymers S6**
3. **Gel Permeation Chromatography S8**
4. **UV-vis absorption and fluorescence spectra S9**
5. **Target analytes S10**
6. **Preparation of polymer films S11**
7. **PFAS acids vapor-phase sensing with polymer films S11**
   - **7.1. Exposure to TFA S12**
   - **7.2. Exposure to TFA of hydrophobic and fluorophilic polymers S12**
   - **7.3. Exposure to PFBA S13**
   - **7.4. Exposure to GenX S13**
   - **7.5. Exposure to PFBS S14**
   - **7.6. Exposure to non-fluorous acid compounds S14**
   - **7.7. Calibration curve of PFBS and PFOA S15**
   - **7.8. Film thickness effect during PFAS acids vapor detection S15**
8. **NMR spectra S16**

1. Materials and instruments

All reactions were carried out under argon using oven-dried glassware. Flash column chromatography was carried out using silica gel (high-purity grade, pore size 60 Å, 230-400 mesh particle size, Sigma Aldrich). Commercial reagents and solvents were purchased from Sigma Aldrich, SynQuest, Alfa Aesar, Ambeed or VWR, and were used without further purification unless otherwise noted below.

^{1}H, ^{19}F and ^{13}C NMR spectra were recorded using a three-channel Bruker Avance Neo spectrometer operating at 500.34 MHz and a two-channel Bruker Avance-III HD Nanobay spectrometer operating at 400.13 MHz. High resolution mass spectra (HRMS) were obtained using a JEOL AccuTOF 4G equipped with an ionSense direct-analysis-in-real-time (DART) source, or a Bruker Autoflex Speed matrix-assisted laser desorption/ionization time-of-flight (MALDI-TOF) spectrometer. Molecular weight and dispersity of the polymers was obtained using an Agilent 1260 Infinity system equipped with an Agilent PLgel guard column (5 μm; 50 x 7.5 mm) and three Agilent PLgel analytical columns (5 μm; 300 x 7.5 mm; 105, 104, and 103 Å pore sizes). The instrument was calibrated with polystyrene standards between 1.7 and 3150 kg mol^{-1}. All samples were prepared in HPLC-grade tetrahydrofuran. Each run was performed at a 1.0 mLmin^{-1} flow rate and 35 °C. Molecular weight values were calculated using ChemStation GPC Data Analysis Software (rev. B.01.01) based on the refractive index signal.

UV-vis absorption spectra were recorded on a Cary 5000 spectrophotometer and corrected for background signal with a solvent-filled cuvette for solutions and glass slide for thin films. Fluorescence measurements were performed using a Horiba Quanta-ϕ fluorescence spectrophotometer, using right-angle detection for solutions, and right-angle detection with an optical fiber for thin films.

2. Synthesis of monomers and polymers

Dibromofluorene **1**, boronic ester **2**, and polymers **PF-Py*** and **PF-Py** were synthesized following a previously reported procedure (Figure S1).[60]

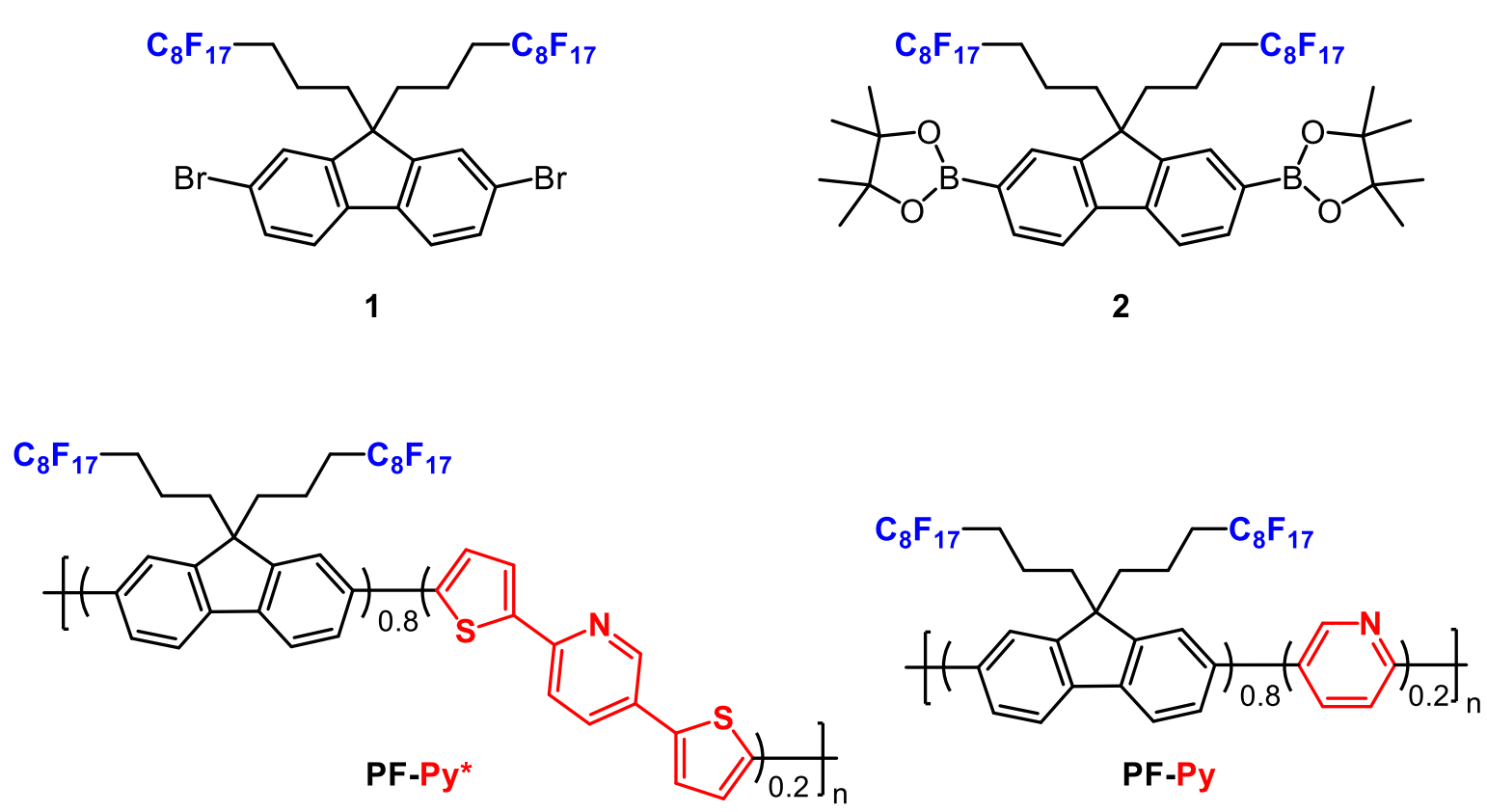

Figure S1. Monomers and polymers previously reported.

2.1. Synthesis of monomers

2.1.1. Compound 4

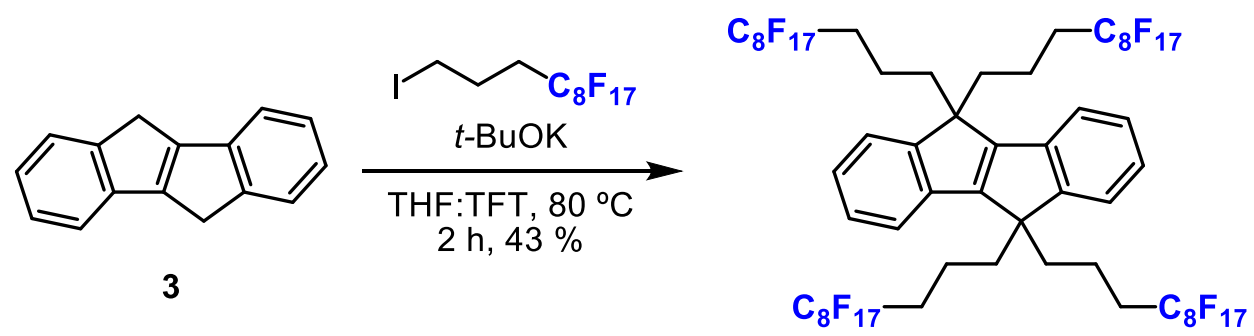

To a solution of 5,10-dihydroindeno[2,1-*a*]indene (**3**, 100 mg, 0.49 mmol) in 10 mL of THF at 0 ºC, *t*-BuOK (440 mg, 3.92 mmol) was added. After 5 minutes, a solution of 3-(perfluorooctyl)propyl iodide (1.44 g, 2.45 mmol) in 6 mL of trifluorotoluene was added dropwise, and the mixture was heated at 80 ºC for 2 hours under argon atmosphere. Then, EtOAc was added to the mixture without cooling down to r.t. and the organic phase was washed with water, brine, and dried over anhydrous sodium sulfate. The solution was filtered, and the solvent was removed under reduced pressure. The product was purified by consecutive washes with EtOH, CH_2Cl_2 and toluene, to afford **4** (430 mg, 43 %) as a pale white solid.

^{1}H NMR (400 MHz, CD_2Cl_2) δ: 7.41-7.31 (m, 6H), 7.27 (td, *J* = 7.1, 1.8 Hz, 2H), 2.26 (td, *J* = 12.7, 4.9 Hz, 4H), 2.14 (td, *J* = 12.6, 4.5 Hz, 4H), 1.95-1.78 (m, 8H), 1.21-1.04 (m, 4H), 1.04-0.82 (m, 4H) ppm. **^{19}F NMR** (471 MHz, $CDCl_3$) δ: -80.91, -80.93, -80.95, -114.66, -114.69, -122.06, -122.11, -122.14, -122.16, -122.18, -122.90, -122.93, -122.95, -122.98, -123.71, -123.74, -123.77, -126.26, -126.28, -126.30, -126.32 ppm. **MS** (MALDI-TOF) *m/z* calculated for $C_{60}H_{32}F_{68}$ $[M]^+$, 2044.1418; found, 2044.1987.

2.1.2. Compound 5

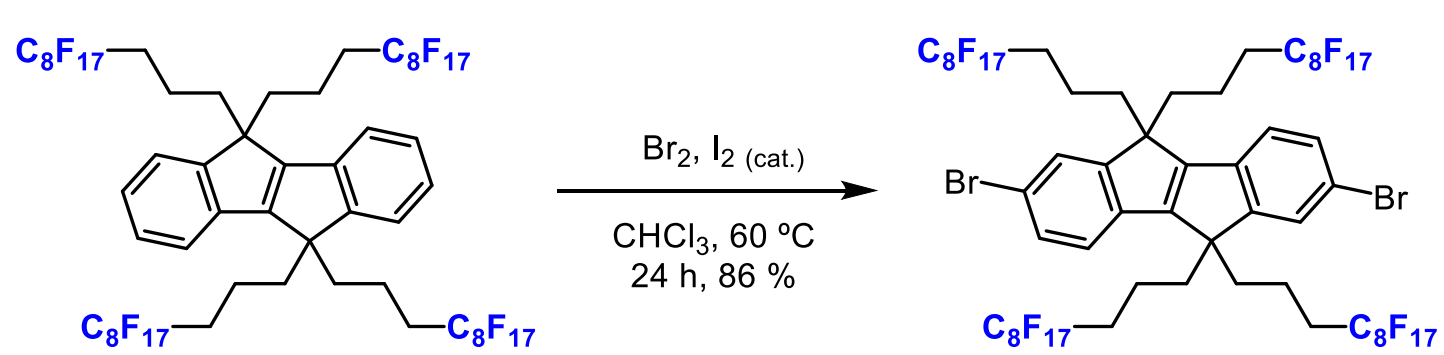

Perfluoroalkylated IndenoIndene **4** (310 mg, 0.152 mmol) was dissolved in $CHCl_3$ (12.5 mL). Then, a teaspoon of I_2 was added to the solution mixture followed by the addition of Br_2 (94 µL, 1.82 mmol) and the mixture was stirred at 60 ºC for 24 h. Then, the reaction was allowed to cool down to r.t., and subsequently the solvent was removed under reduced pressure. The crude product was washed with EtOH and recrystallized from $CHCl_3$ obtaining **5** (288 mg, 86 %) as a white solid.

^{1}H NMR (500 MHz, $CDCl_3$) δ: 7.53-7.44 (m, 4H), 7.18 (d, *J* = 7.9 Hz, 2H), 2.20 (td, *J* = 12.9, 4.7 Hz, 4H), 2.09 (td, *J* = 12.8, 4.4 Hz, 4H), 1.99 – 1.74 (m, 8H), 1.16 – 1.03 (m, 4H), 1.04 – 0.82 (m, 4H) ppm. **^{19}F NMR** (471 MHz, $CDCl_3$) δ: -80.89, -80.91, -80.94, -114.51, -114.71, -121.99, -122.11, -122.13, -122.16, -122.20, -122.91, -122.97, -123.65, -123.69, -123.73, -126.24, -126.27, -126.27, -126.31 ppm. **MS** (MALDI-TOF) *m/z* calculated for $C_{60}H_{30}Br_2F_{68}$ $[M]^+$, 2199.9628; found, 2200.0270.

2.1.3. Compound 6

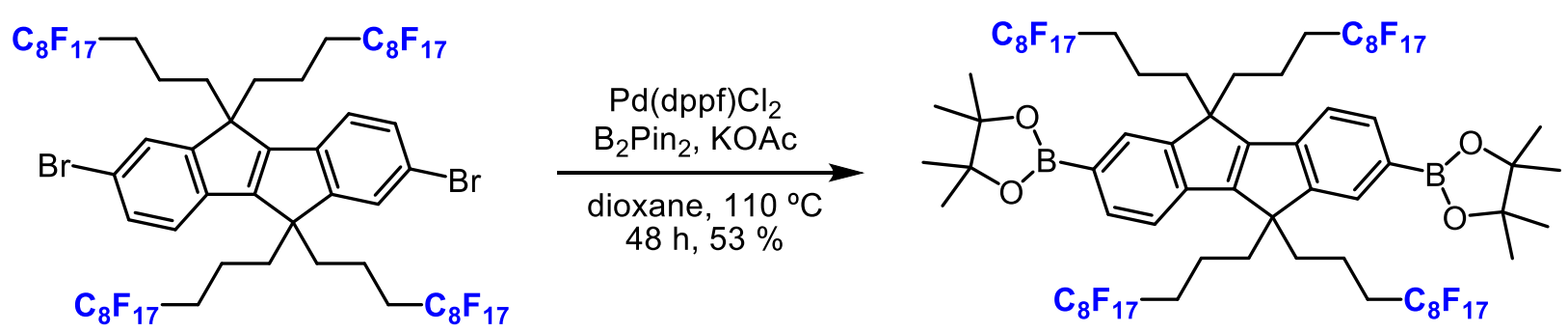

5 6

Perfluoroalkylated dibromoIndenoIndene **5** (200 mg, 0.091 mmol), B_2Pin_2 (69.3 mg, 0.273 mmol), KOAc (53.6 mg, 0.546 mmol) and $Pd(dppf)Cl_2$ (13.3 mg, 0.018 mmol) were dissolved in anhydrous dioxane (8 mL). The mixture was heated at 110 ºC (reflux) for 48 h under argon atmosphere. Then, water was added to the mixture and the precipitate was filtered. The crude product was dissolved in hot $CHCl_3$ and filtered through a silica plug with hot $CHCl_3$ and hot EtOAc. The solvent was removed under reduced pressure and the product was recrystallized from acetone obtaining **6** (110 mg, 53 %) as a pale white solid.

^{1}H NMR (500 MHz, $CDCl_3$) δ: δ 7.83 (d, J = 7.5 Hz, 2H), 7.73 (s, 2H), 7.34 (d, J = 7.4 Hz, 2H), 2.31 – 2.13 (m, 8H), 1.91 – 1.73 (m, 8H), 1.38 (s, 24H), 1.15 – 1.02 (m, 4H), 1.01 – 0.79 (m, 4H) ppm. **^{19}F NMR** (471 MHz, $CDCl_3$) δ: -80.91, -80.93, -80.96, -114.60, -114.77, -114.84, -122.03, -122.15, -122.16, -122.19, -122.23, -122.96, -123.70, -123.74, -123.78, -126.26, -126.29, -126.30, -126.33 ppm. **MS** (MALDI-TOF) *m/z* calculated for $C_{72}H_{54}B_2F_{68}O_4$ $[M]^+$, 2296.3122; found, 2296.3832.

2.1.4. Compound 8

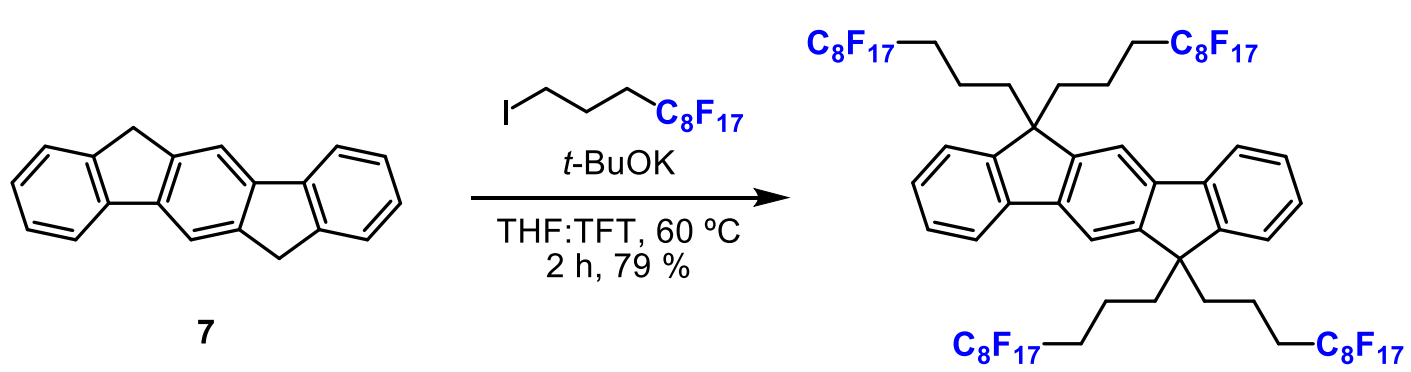

7 8

To a solution of 6,12-dihydroindeno[1,2-*b*]fluorene (**7**, 0.100 g, 0.393 mmol) in 10 mL of THF at 0 ºC, *t*-BuOK (353 mg, 3.14 mmol) was added. After 5 minutes, a solution of 3-(perfluorooctyl)propyl iodide (1.15 g, 1.95 mmol) in 3 mL of trifluorotoluene was added dropwise, and the mixture was heated at 60 ºC for 2 hours under argon atmosphere. Then, the mixture was allowed to cool down to r.t., poured into water and extracted three times with EtOAc. The combined organic phases were washed with water, brine and dried over anhydrous sodium sulfate. The solution was filtered, and the solvent was removed under reduced pressure. The product was purified by column chromatography (SiO_2, EtOAc/hexanes, 5/95) obtaining **8** (0.650 g, 79 %) as white solid.

^{1}H NMR (500 MHz, $CDCl_3$) δ: 7.78 (d, J = 7.4 Hz, 2H), 7.65 (s, 2H), 7.44 – 7.37 (m, 2H), 7.37 – 7.31 (m, 4H), 2.17 (t, J = 8.0 Hz, 8H), 1.87 – 1.69 (m, 8H), 1.02 – 0.83 (m, 8H) ppm. **^{19}F NMR** (471 MHz, $CDCl_3$) δ: -80.90, -80.92, -80.94, -114.78, -114.82, -114.85, -122.01, -122.05, -122.16, -122.18, -122.20, -122.94, -123.77, -123.82, -123.85, -126.25, -126.28, -126.30, -126.33 ppm.

2.1.5. Compound 9

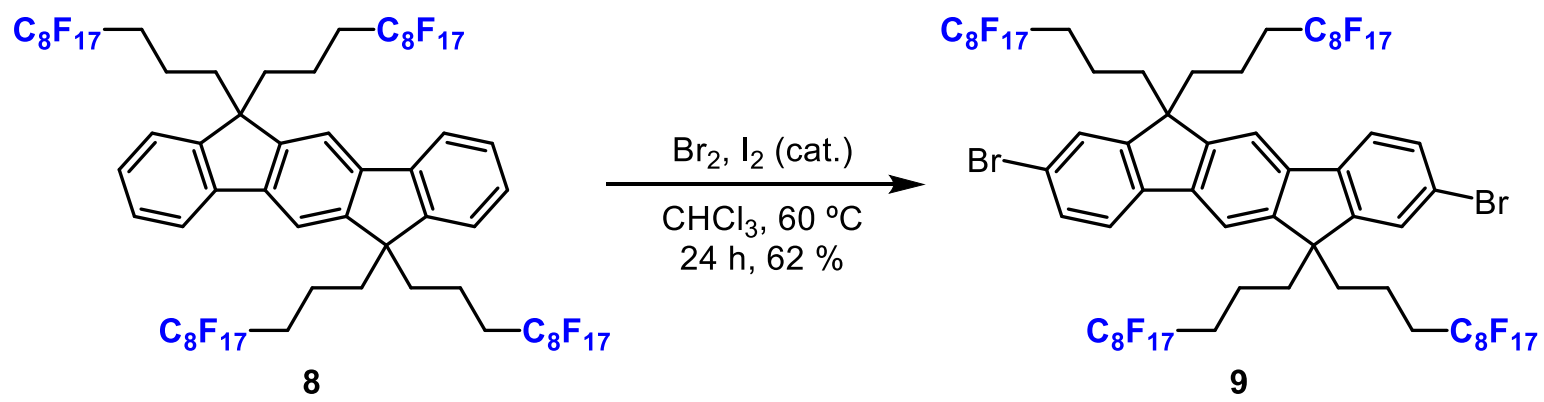

Perfluoroalkylated IndenoFluorene **8** (200 mg, 0.095 mmol) was dissolved in $CHCl_3$ (10 mL). Then, a teaspoon of I_2 was added to the solution mixture followed by the addition of Br_2 (40 µL, 0.764 mmol) and the mixture was stirred at 60 ºC for 6 h. After this time, another teaspoon of I_2 was added followed by the addition of Br_2 (40 µL, 0.764 mmol) and the mixture was stirred at 60 ºC for 16 h. Next day, the resulting mixture was washed with an aqueous solution of $Na_2S_2O_3$, and the aqueous layer was extracted with large quantities of hot $CHCl_3$ (at least three times). The combined organic phases were washed with water, brine and then solvent was removed under reduced pressure. The product was purified by several washed with EtOH to afford **9** (116 mg, 54 %) as a light-yellow solid.

^{1}H NMR (500 MHz, $CDCl_3$) δ: 7.65 (d, *J* = 8.1 Hz, 2H), 7.61 (s, 2H), 7.54 (dd, *J* = 8.1, 1.8 Hz, 2H), 7.49 (d, *J* = 1.8 Hz, 2H), 2.23 – 2.07 (m, 8H), 1.89 – 1.68 (m, 8H), 0.98 – 0.81 (m, 8H) ppm. **^{19}F NMR** (471 MHz, $CDCl_3$) δ: -80.88, -80.91, -80.93, -114.69, -114.73, -114.76, -114.80, -114.84, -122.03, -122.10, -122.12, -122.14, -122.16, -122.18, -122.21, -122.92, -123.76, -126.25, -126.27, -126.31 ppm.

2.1.6. Compound 11

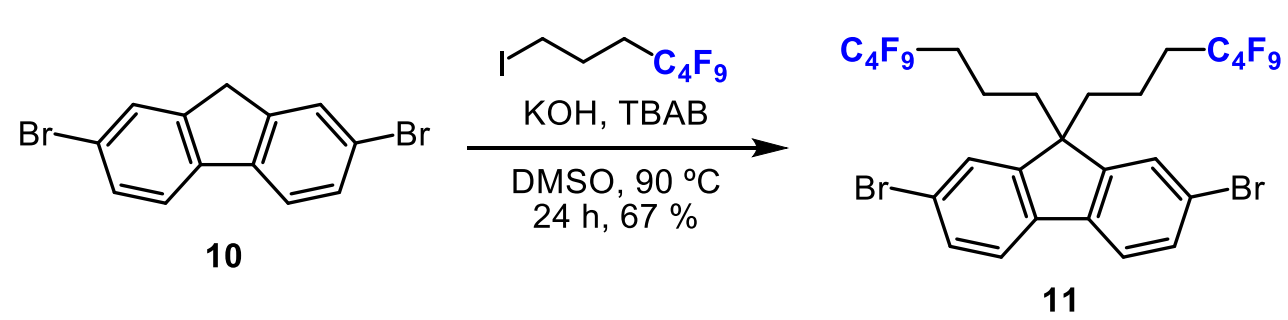

2,7-dibromofluorene (**10**, 0.692 g, 2.14 mmol) and 3-(perfluorobutyl)propyl iodide (1.83 g, 4.71 mmol), tetrabutylammonium bromide (70 mg, 0.214 mmol) were dissolved in DMSO (15 mL) and heated to 90 ºC. Subsequently, an aqueous solution of potassium hydroxide (2.40 g, 42.8 mmol, in 5 mL H_2O) was added dropwise to the reaction flask and the reaction mixture was stirred at 90 ºC for 24 h under argon atmosphere. Then, the mixture was allowed to cool down to r.t., poured into water and extracted three times with EtOAc. The combined organic phases were washed with water, brine and dried over anhydrous magnesium sulfate. The solution was filtered, and the solvent was removed under reduced pressure. The product was purified by column chromatography (SiO_2, Hexanes) obtaining **11** (1.21 g, 67 %) as a colorless oil.

^{1}H NMR (500 MHz, $CDCl_3$) δ: 7.57 (d, *J* = 8.1 Hz, 2H), 7.52 (dd, *J* = 8.1, 1.8 Hz, 2H), 7.46 (d, *J* = 1.7 Hz, 2H), 2.13 – 2.00 (m, 4H), 1.92 – 1.76 (m, 4H), 1.00 – 0.83 (m, 4H) ppm. **^{19}F NMR** (471 MHz, $CDCl_3$) δ: -81.06, -81.08, -81.10, -114.59, -114.63, -114.67, -124.71, -124.73, -124.75, -124.77, -126.05, -126.08, -126.09, -126.11 ppm. **HRMS** (DART) *m/z* calculated for $C_{27}H_{18}Br_2F_{18}$ $[M]^+$, 841.94823; found, 841.94526.

2.1.7. Compound 12

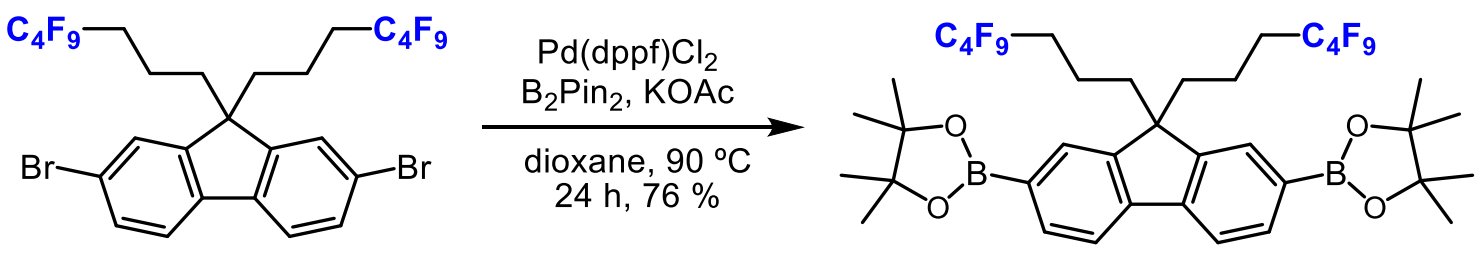

Compound **11** (500 mg, 0.592 mmol), B_2Pin_2 (376 mg, 1.48 mmol), KOAc (349 mg, 3.55 mmol) and $Pd(dppf)Cl_2$ (48 mg, 0.0592 mmol) were dissolved in anhydrous dioxane (10 mL). The mixture was heated at 90 ºC for 24 h under argon atmosphere. Then, water was added to the reaction mixture, and the product was extracted three times with ethyl acetate. The combined organic phases were washed with water, brine and dried over anhydrous magnesium sulfate. The solution was filtered, and the solvent was removed under reduced pressure. The product was purified by column chromatography (SiO_2, EtOAc/hexanes, 5/95) obtaining **12** (422 mg, 76 %) as a white solid.

^{1}**H NMR** (500 MHz, $CDCl_3$) δ: 7.85 (d, *J* = 7.5 Hz, 2H), 7.78 – 7.71 (m, 4H), 2.26 – 2.06 (m, 4H), 1.84 – 1.67 (m, 4H), 1.39 (s, 24H), 0.99 – 0.77 (m, 4H) ppm. ^{19}**F NMR** (471 MHz, $CDCl_3$) δ: -81.09, -81.11, -81.13, -114.65, -114.69, -114.73, -124.77, -124.79, -124.81, -124.83, -126.12 ppm. **HRMS** (DART) *m/z* calculated for $C_{39}H_{42}B_2F_{18}O_4$ $[M]^+$, 938.29763; found, 938.29934.

2.2. Synthesis of polymers
2.2.1. General procedure

A degassed benzotrifluoride solution (0.08 M) was added to a Schlenk flask containing dihaloarenes, boronic esters, and tetrakis(triphenylphosphine)palladium (0). Then, an aqueous solution of tetraethylammonium hydroxide (35 wt. % in water, 0.08 M) was added. The flask was deoxygenated by three freeze−pump−thaw cycles and flushed with argon. The reaction mixture was stirred at 90 °C for 48 hours. After cooling down to r.t., the mixture was precipitated into methanol. The solid was re-dissolved in benzotrifluoride and precipitated in methanol two times followed by a final precipitation in acetone.

2.2.2. Polymer PII-Py

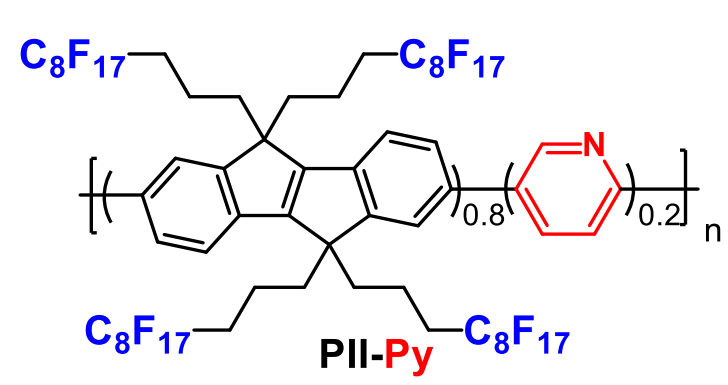

PII-Py was prepared following the general procedure using dibromo **5** (28.85 mg, 0.0131 mmol), boronic ester **6** (50.00 mg, 0.0218 mmol), 2,5-dibromopyridine (2.06 mg, 0.00872 mmol), and $Pd(PPh_3)_4$ (2.52 mg, 0.00218 mmol) to afford the polymer as a green solid (86 %) with low solubility in $CHCl_3$ and THF. ^{1}**H NMR** (400 MHz, $CDCl_3$) δ: 9.65 – 9.36 (s, 0.20H), 8.54 – 8.28 (m, 0.51H), 8.13 – 7.93 (m, 0.72H), 7.86 – 7.38 (m, 4.69H), 2.50 – 2.22 (m, 4.47H), 2.15 – 1.63 (m, 16H), 1.49 – 0.95 (m, 5.36H) ppm. ^{19}**F NMR** (376 MHz, $CDCl_3$) δ: -81.08, -114.76, -122.17, -122.99, -123.69, -124.00, -126.39 ppm. **GPC** (THF): M_n = 3.57 kDa, $Đ$ = 1.12.

2.2.3. Polymer PIF-PF-Py

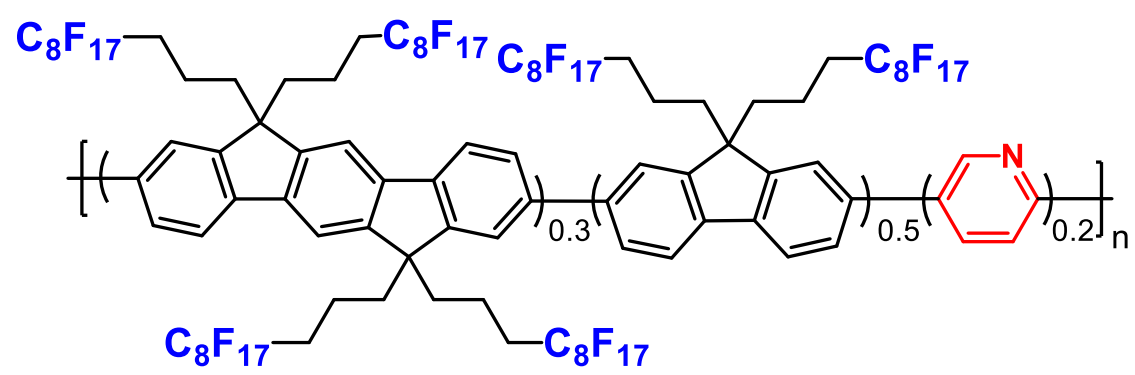

PIF-PF-Py

PIF-PF-Py was prepared following the general procedure using dibromo **9** (81.1 mg, 0.036 mmol), boronic ester **2** (80.3 mg, 0.060 mmol), 2,5-dibromopyridine (5.70 mg, 0.024 mmol), and $Pd(PPh_3)_4$ (7.00 mg, 0.006 mmol) to afford the polymer as a dark green solid (72 %) with low solubility in $CHCl_3$ and THF. **^{1}H NMR** (400 MHz, $CDCl_3$)) δ: 9.05 (s, 0.20H), 8.25 – 8.01 (m, 0.83H), 7.98 – 7.84 (m, 0.64H), 7.81 – 7.35 (m, 1.90H), 2.46 – 2.11 (m, 1.63H), 2.00 – 1.71 (m, 1.60H), 1.36 – 0.94 (m, 1.56H) ppm. **^{19}F NMR** (376 MHz, $CDCl_3$) δ: -80.83, -80.92, -81.06, -114.35, -122.06, -122.87, -123.88, -126.26 ppm. **GPC** (THF): M_n = 6.08 kDa, $Đ$ = 1.18.

2.2.4. Polymer C_8/C_4PF-Py

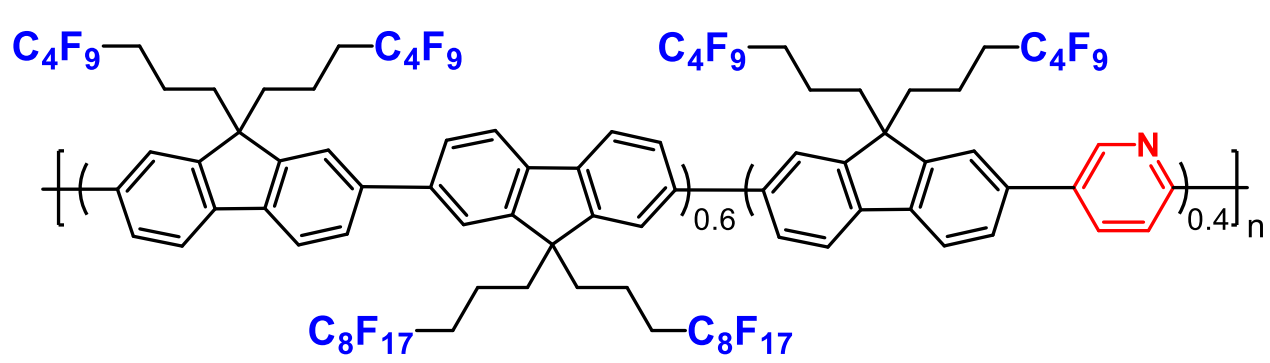

C_8/C_4PF-Py

C_8/C_4PF-Py was prepared following the general procedure using dibromo **1** (120 mg, 0.096 mmol), boronic ester **12** (150 mg, 0.160 mmol), 2,5-dibromopyridine (15.0 mg, 0.064 mmol), and $Pd(PPh_3)_4$ (9.00 mg, 0.008 mmol) to afford the polymer as a light green solid (71 %). **^{1}H NMR** (400 MHz, $CDCl_3$) δ: 9.06 (s, 0.40H), 8.23 – 8.03 (m, 1.35H), 8.02 – 7.46 (m, 13H), 2.43 – 2.04 (m, 8H), 2.01 – 1.71 (m, 8H), 1.34 – 0.95 (m, 8H) ppm. **^{19}F NMR** (376 MHz, $CDCl_3$) δ: -80.91, -81.30, -81.44, -114.41, -114.69, -121.89, -122.07, -122.86, -123.96, -124.88, -125.00, -126.23, -126.33 ppm. **GPC** (THF): M_n = 9.62 kDa, $Đ$ = 1.59.

2.2.5. Polymer AlkylPF-Py

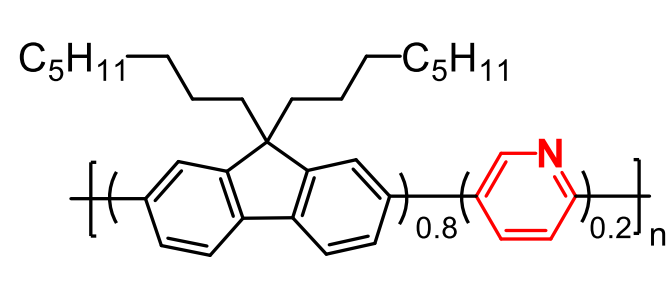

AlkylPF-Py

C_8/C_4PF-Py was prepared following the general procedure using 9,9-Dioctyl-2,7-dibromofluorene (240 mg, 0.438 mmol), 9,9-Dioctylfluorene-2,7-diboronic acid bis(1,3-propanediol) ester (407 mg, 0.730 mmol), 2,5-dibromopyridine (69.1 mg, 0.292 mmol), $Pd(PPh_3)_4$ (4.20 mg, 0.0036 mmol) and toluene as solvent to afford the polymer as a green solid (92 %). **^{1}H NMR** (400 MHz, $CDCl_3$) δ: 9.09 (s, 0.2H), 8.24 – 8.04 (m, 0.6H), 8.01 – 7.31 (m, 5H), 2.26 – 1.90 (m, 2H), 1.27 – 0.99 (m, 14H), 0.95 – 0.64 (m, 6H) ppm. **GPC** (THF): M_n = 17.5 kDa, $Đ$ = 2.27

3. Gel Permeation Chromatography

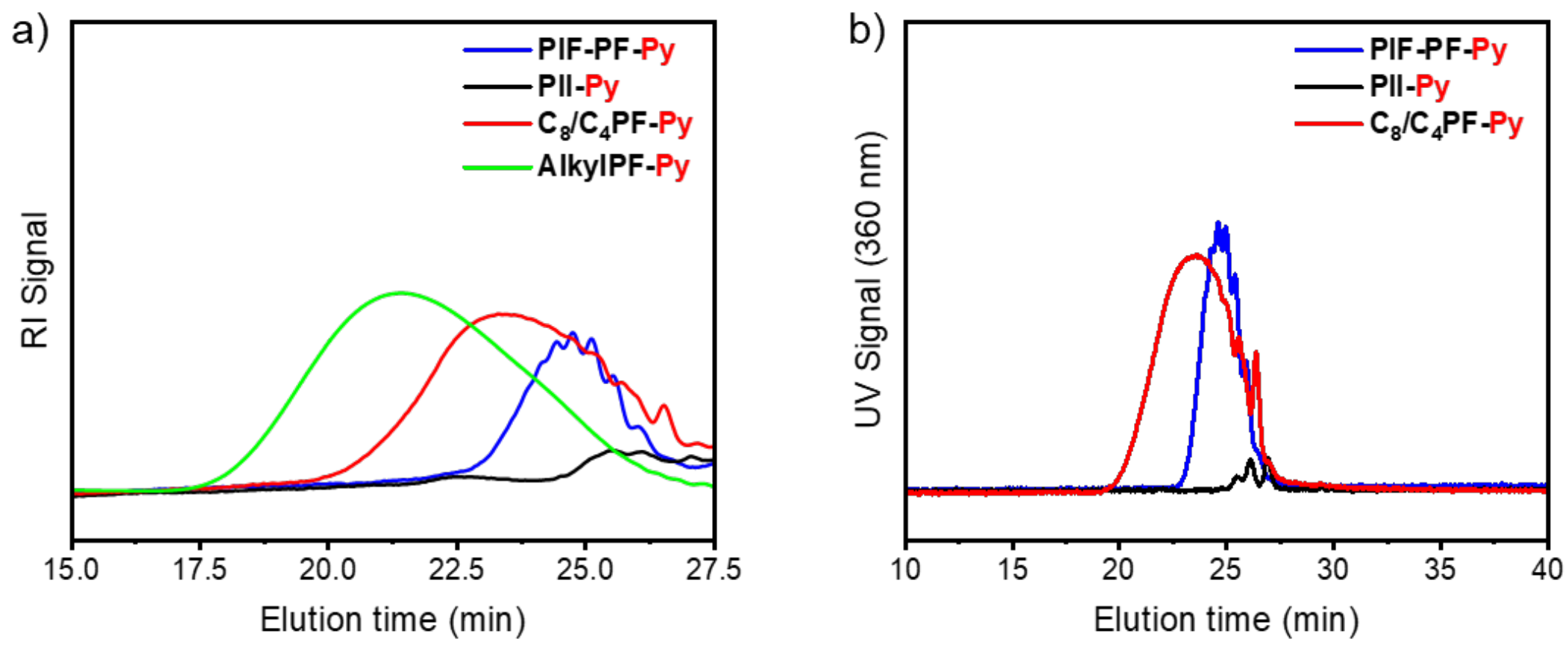

Figure S2. GPC traces of **PIF-PF-Py**, **PII-Py**, **C_8/C_4PF-Py**, and **AlkylPF-Py**. a) Refractive Index signal, and b) UV signal (360 nm).

Table S1. Summary of the weight % fluorine (wt F %), molecular weight (M_n) and polydispersity (*Đ*) of polymers used in this work.

Polymer	wt F %[a]	M_n	*Đ*
**PF-Py***[60]	56.4	8.90 kDa	2.06
**PF-Py**[60]	58.5	8.10 kDa	1.88
**PIF-PF-Py**[b]	59.9	6.08 kDa	1.18
**PII-Py**[b]	62.6	3.57 kDa	1.12
**C_8/C_4PF-Py**	53.3	9.62 kDa	1.59
**AlkylPF-Py**	0	17.5 kDa	2.27

[a]Fluorine content of the repeating unit. [b]Not very soluble in the GPC mobile phase (THF).

4. UV-vis absorption and fluorescence spectra

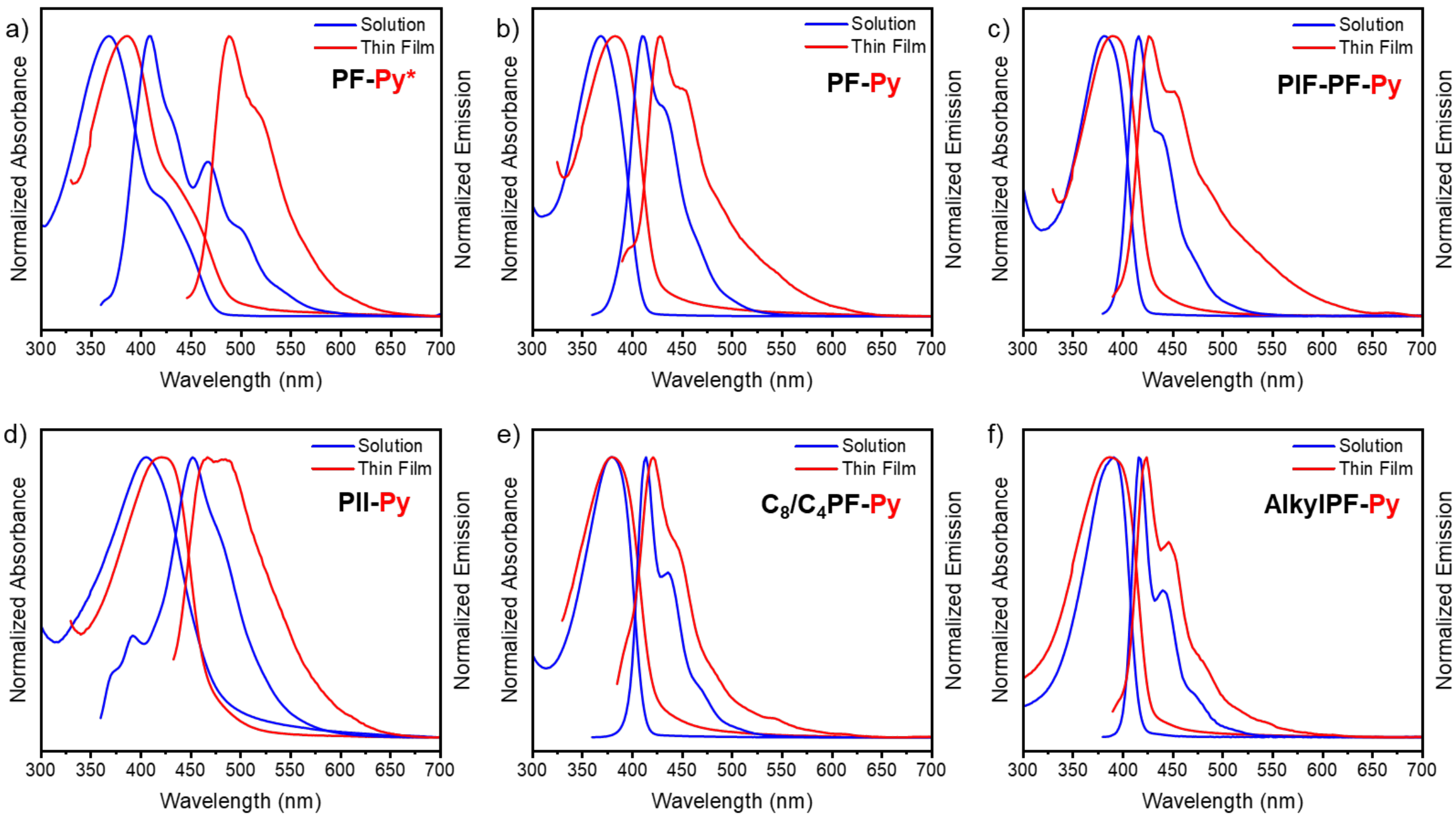

Figure S3. UV-vis and fluorescence spectra in THF solutions, and spin-cast films: a) **PF-Py***, b) **PF-Py**, c) **PIF-PF-Py**, d) **PII-Py**, e) **C_8/C_4PF-Py**, and f) **C_8/C_4PF-Py**

Table S2. Optical properties of the polymers used in this work.

Polymer	Absorption (λ_{max})		Emission (λ_{max})	
	THF	Thin Film	THF	Thin Film
**PF-Py***	368 nm	387 nm	408 nm	488 nm
**PF-Py**	369 nm	383 nm	410 nm	428 nm
**PIF-PF-Py**	381 nm	390 nm	416 nm	430 nm
**PII-Py**	406 nm	421 nm	452 nm	462 nm
**C_8/C_4PF-Py**	380 nm	380 nm	414 nm	419 nm
**AlkylPF-Py**	391 nm	387 nm	416 nm	424 nm

5. Target analytes

The vapor-phase concentrations were calculated using controlled headspace conditions. A 3 mL volume in the headspace was established by placing 1 mL of analyte in a 4 mL vial, which was subsequently enclosed within a sealed 20 mL vial.

The experiments were carried out at 25 ºC, and this temperature was used in all calculations. When vapor pressure data were only available at other temperatures, the associated uncertainty was considered to fall within the experimental error of the calculated concentrations.

$$\mathrm{P_i} = X_\mathrm{i} \cdot \mathrm{P_i}^{\circ} \qquad \text{Equation (1)}$$

$\mathrm{P_i}$: Partial vapor pressure of i; X_i: Mole fraction of i; $\mathrm{P_i}^{\circ}$**:** Absolute vapor pressure of i

Vapor-phase concentrations in the headspace were calculated using Raoult's law (Equation 1) in combination with the ideal gas law (Equation 2). For the pure compounds, the absolute vapor pressure was used together with the ideal gas law to calculate the vapor-phase mass concentration in the headspace. Alternatively, vapor-phase concentrations can be expressed as part per million by volume using the ratio of the partial pressure of the compound in the headspace to the total pressure (Equation 3). Under ideal gas assumptions, this expression is equivalent to the conversion from mass concentration to $\mathrm{ppm_v}$.

$$\mathrm{P_i\,V = nRT} \rightarrow \mathrm{C} = \frac{\mathrm{P_i}}{\mathrm{RT}} \rightarrow \mathrm{C} = \frac{\mathrm{P_i \cdot Mw \cdot 1000}}{\mathrm{RT}} \qquad \text{Equation (2)}$$

$\mathrm{P_i}$: Partial vapor pressure of i (Pa); Mw: Molecular Weight of i; R: Gas constant (8.314 J/molK); T**:** Temperature (298 K); C**:** Concentration in the headspace (mg/m^3)

$$\mathrm{ppm_v} = \frac{\mathrm{P_i}}{\mathrm{P_T}} \cdot 10^6 \qquad \text{Equation (3)}$$

$\mathrm{P_i}$: Partial vapor pressure of i (Pa); $\mathrm{P_T}$: Total pressure (101325 Pa)

PFAS acids solutions were prepared in chloroform with a total volume of 1 mL, and the partial vapor pressure of each analyte was calculated from its mole fraction in solution and the vapor pressure of the pure compound using Raoult's law (Equation 1). Chloroform was selected as the solvent due to negligible interaction with PFAS acids, minimizing non-ideal gas behavior. Corresponding headspace concentrations were calculated assuming ideal gas behavior. Systematic dilution of the solutions enabled precise control over analyte concentration in the vapor phase.

Table S3. Compounds used during sensing studies, including reported vapor pressure values and calculated concentrations in the headspace.

Compound	Mw (g/mol)	Density (g/mL)	Vapor pressure	[Vapor phase]
**TFA**	114.02	1.489	97.5 mmHg (20ºC)[a]	128289 ppm<br>598 g/m^3
**PFBA**	214.04	1.645	10 mmHg (25ºC)[a]	13158 ppm<br>115 g/m^3
**GenX**	330.05	1.690	2.7 mmHg (20ºC)[b]	3553 ppm<br>47.9 g/m^3
**PFBS**	300.10	1.811	7 Pa =<br>0.0525 mmHg (20ºC)[c]	69 ppm<br>0.85 g/m^3
**PFOA**	414.07	1.792	0.0316 mmHg (19ºC)[d]	41.6 ppm<br>0.70 g/m^3
**AcOH**	60.050	1.049	11.4 mmHg (20ºC)[d]	15000 ppm<br>36.8 g/m^3
**BA**	88.110	0.964	0.43 mmHg (20ºC)[a]	566 ppm<br>2.04 g/m^3
**OA**	144.21	0.910	0.05 hPa =<br>0.0375 mmHg (20ºC)[a]	49.3 ppm<br>0.29 g/m^3
**FA**	46.030	1.220	44.8 mmHg (20 ºC)[a]	58947 ppm<br>111 g/m^3

[a]Sigma Aldrich; [b]i) W. Guo, W. Hao, W. Xiao Environ. Health 2025, 3, 338−351. ii) EPA-823-P-18-001; [c]i) Michigan Department of Environment. ii) REACH, 2018; [d]Pubchem.

6. Preparation of polymer films

Glass substrates were cleaned by sequential sonication in soapy water, milli-Q water, isopropanol, and followed by UV-ozone treatment. Then, the glass substrates were immersed in a toluene solution containing 1% (v/v) hexamethyldisilazane (HMDS) and dried in an oven at 110 ºC for 1 hour. Polymers were dissolved in THF at concentration of 1 mg/mL and filtered using a 0.45 µm filter before applying to the glass substrate. Polymer solutions were deposited on a cover glass (10 x 10 mm) and spin-casted by a WS-400-6NPP/LITE Spin Processor (Laurell Technologies), using a spin rate of 2000 rpm for 30 seconds. Finally, the polymer thin films were placed under vacuum overnight before use.

7. PFAS acids vapor-phase sensing with polymer films

Polymer response studies for TFA, PFBA, GenX, and PFBS were conducted using polymeric thin films, with fluorescence spectra recorded before and after exposure using a bifurcated fiber optic accessory. Ratiometric fluorescence detection was employed to obtain higher accuracy and reliability during the measurements, monitoring both initial polymer emission maxima and the red-

shifted emission maxima induced upon polymer activation. Fluorescence was analyzed immediately after exposing thin films to PFAS acids vapor, recording the emission at defined time intervals (15, 60, 300, 600, and 900 seconds). The fluorescence studies on **PF-Py***, **PF-Py**, **PIF-PF-Py**, **PII-Py**, and **C_8/C_4PF-Py** were performed with excitation wavelengths of 390, 370, 370, 370, and 370 nm, respectively. All experiments were performed in triplicate using three independent polymer films for each analyte and concentration. Controlled headspace conditions were established by placing 1 mL of analyte in a 4 mL vial, which was then enclosed within a sealed 20 mL vial. After vapor pressure equilibration for 15 minutes, the vial was opened, and polymer films were exposed to the vapor for 5 seconds.

7.1. Exposure to TFA

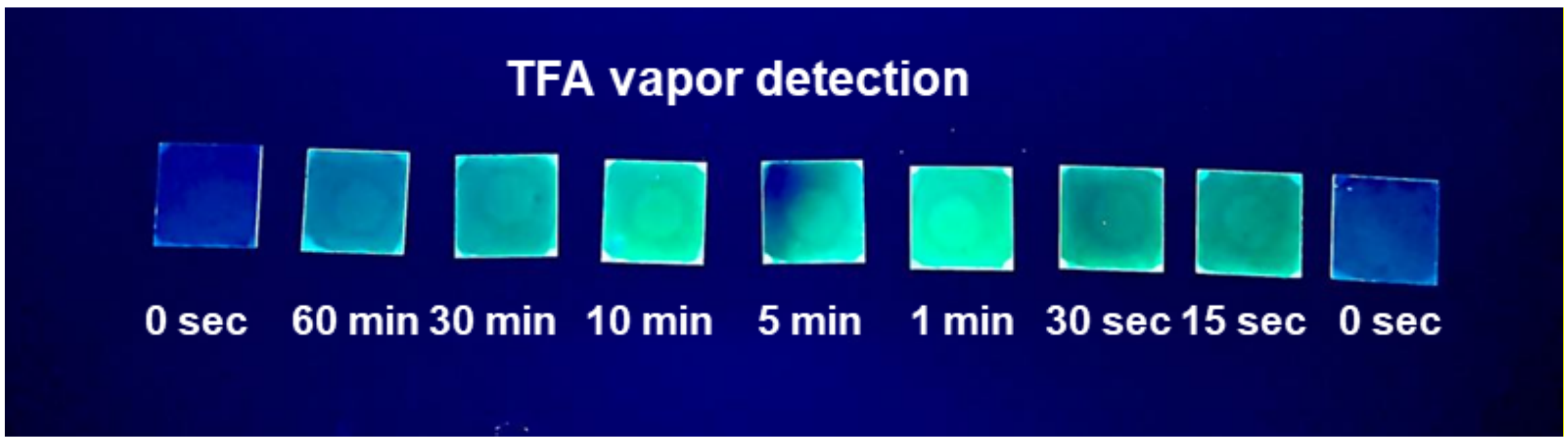

Figure S4. Pictures showing the evolution of the color change of **C_8/C_4PF-Py** thin films after the exposure to TFA vapor for 5 seconds. Visible activation to the naked eye after under irradiation with 365 nm light.

7.2. Comparation of hydrophobic and hydrophilic interactions.

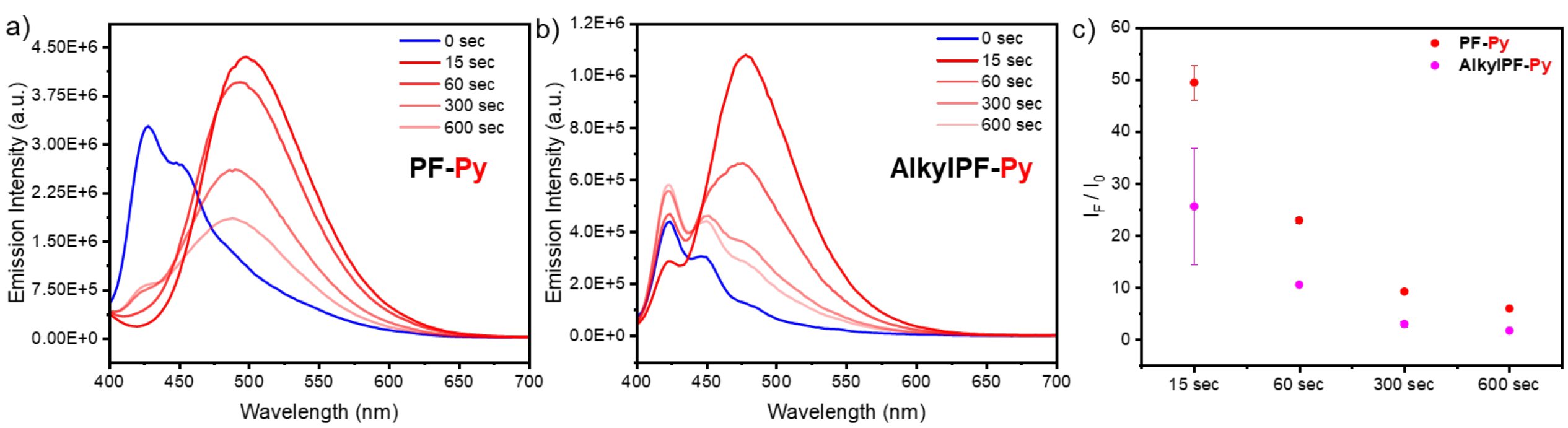

Figure S5. The unactivated polymer emission is shown in blue. Red spectra represent fluorescence recovery over time, with each trace corresponding to a different post-exposure time (increasing transparency indicates longer times). Results after a 5 second exposure to TFA vapor (128289 ppm) using: a) **PF-Py**, b) **AlkylPF-Py**, and c) Plot showing the difference of the final and initial ratiometric fluorescence signal as a function of time. Wavelenghts monitored for **PF-Py** (428 and 498 nm) and **AlkylPF-Py** (424 and 486 nm).

7.3. Exposure to PFBA

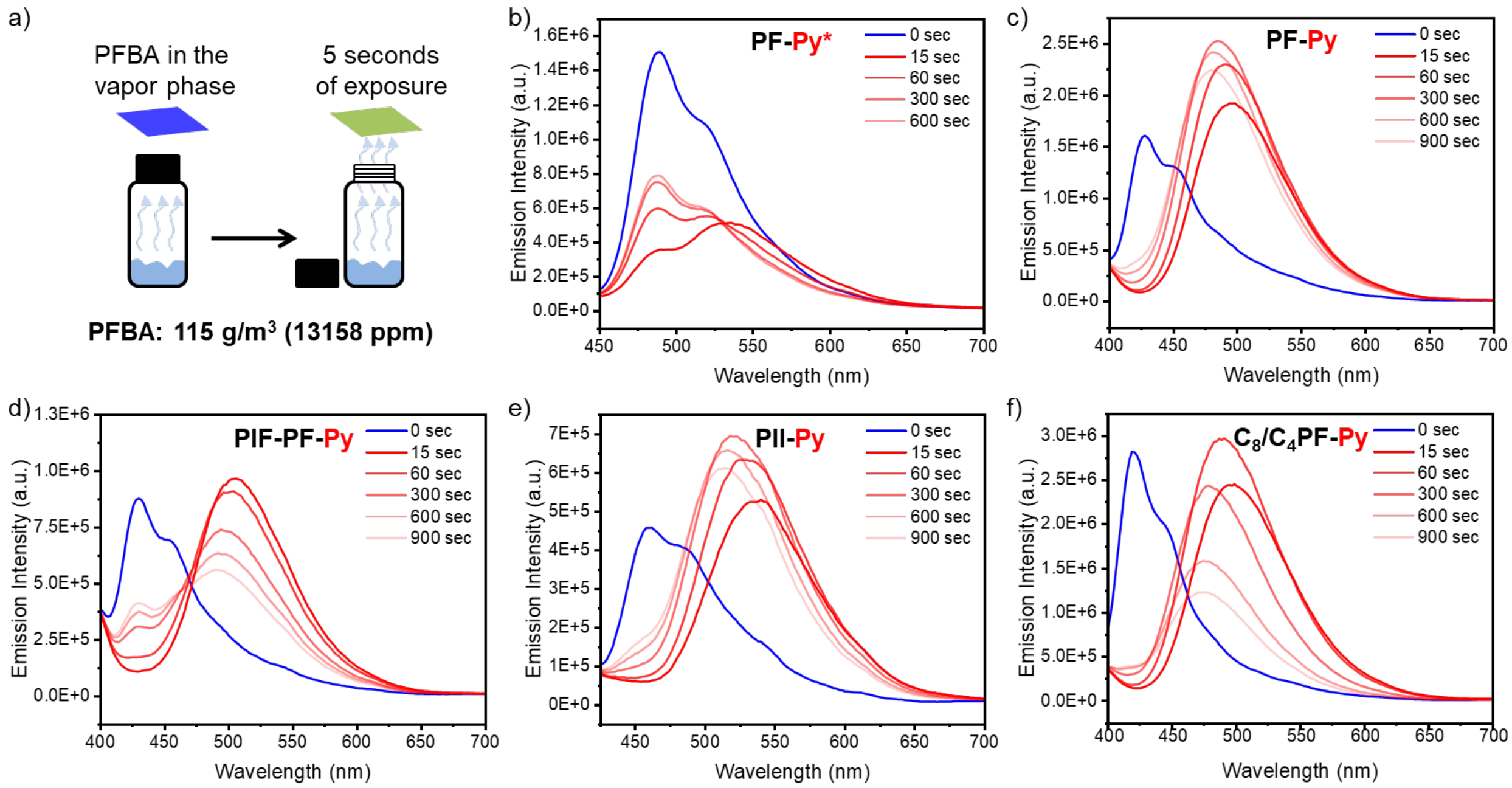

Figure S6. a) Graphical abstract of polymer thin films exposure to PFAS acids in the vapor phase. The unactivated polymer emission is shown in blue. Red spectra represent fluorescence recovery over time, with each trace corresponding to a different post-exposure time (increasing transparency indicates longer times). Results after a 5 second exposure to PFBA vapor using: b) **PF-Py***, c) **PF-Py**, d) **PIF-PF-Py**, e) **PII-Py**, and f) **C_8/C_4PF-Py**.

7.4. Exposure to GenX

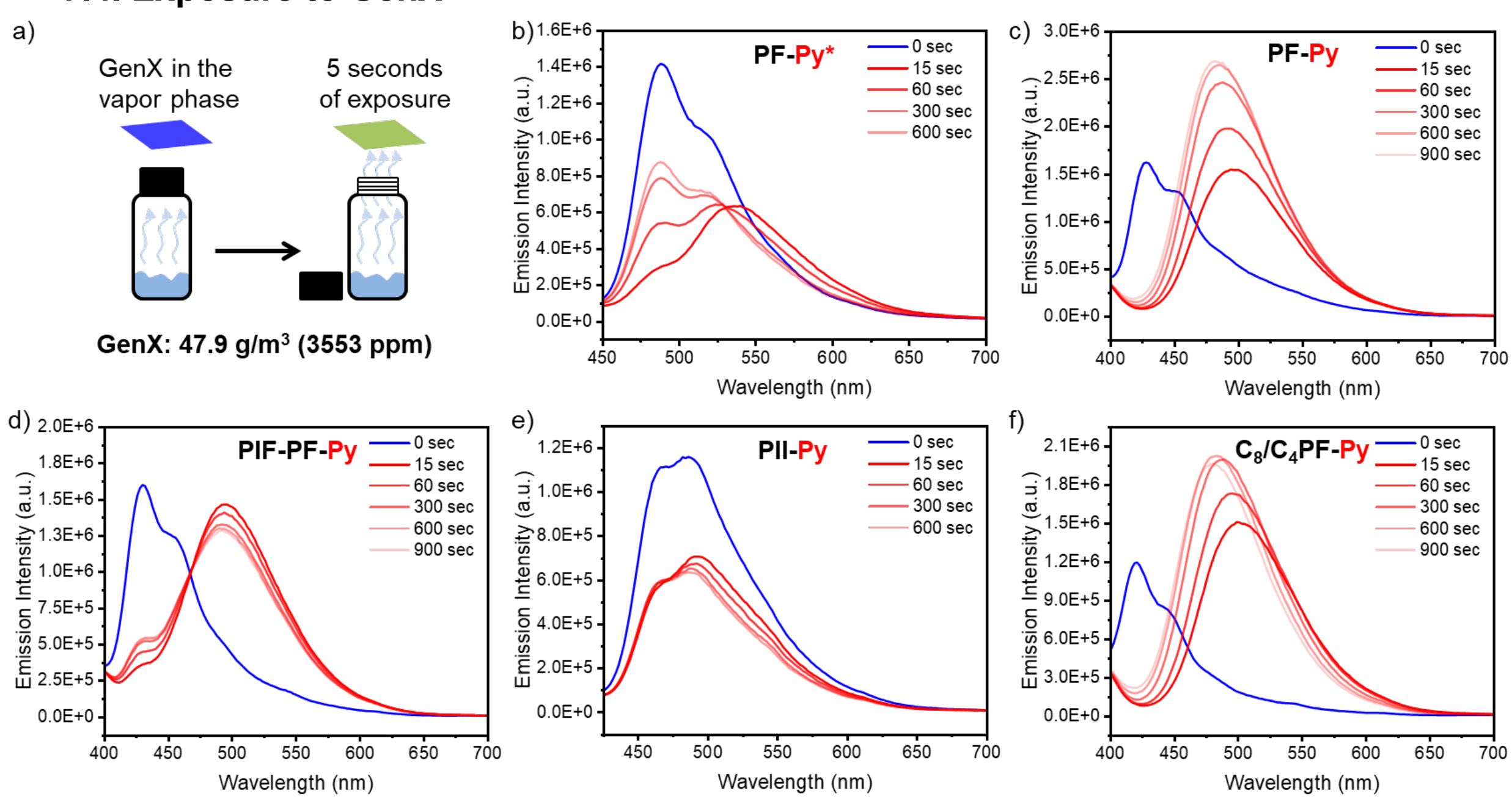

Figure S7. a) Graphical abstract of polymer thin films exposure to PFAS acids in the vapor phase. The unactivated polymer emission is shown in blue. Red spectra represent fluorescence recovery over time, with each trace corresponding to a different post-exposure time (increasing transparency indicates longer times). Results after a 5 second exposure to GenX vapor using: b) **PF-Py***, c) **PF-Py**, d) **PIF-PF-Py**, e) **PII-Py**, and f) **C_8/C_4PF-Py**.

7.5. Exposure to PFBS

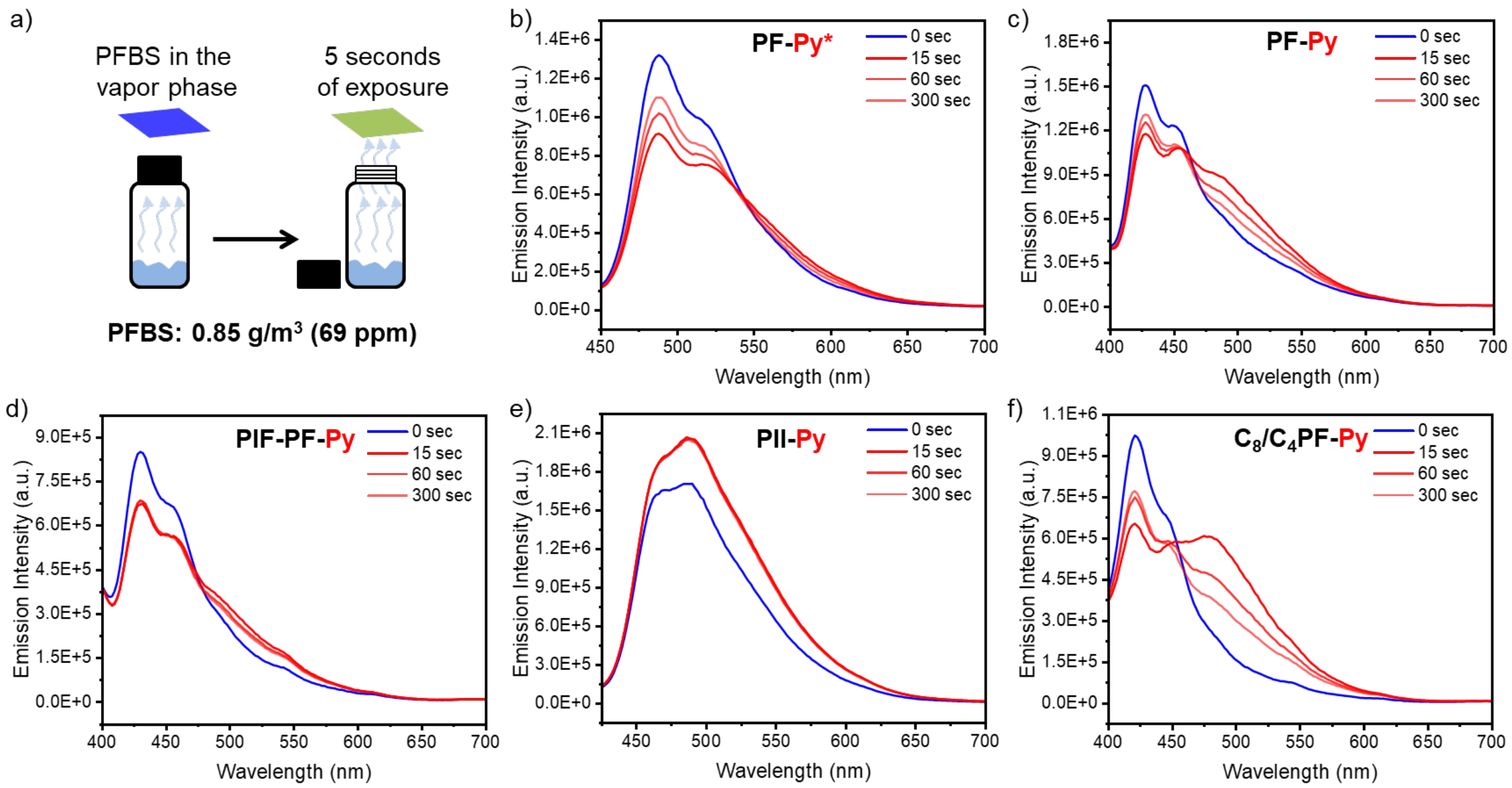

Figure S8. a) Graphical abstract of polymer thin films exposure to PFAS acids in the vapor phase. The unactivated polymer emission is shown in blue. Red spectra represent fluorescence recovery over time, with each trace corresponding to a different post-exposure time (increasing transparency indicates longer times). Results after a 5 second exposure to PFBS vapor using: b) **PF-Py***, c) **PF-Py**, d) **PIF-PF-Py**, e) **PII-Py**, and f) **C_8/C_4PF-Py**.

7.6. Exposure to non-fluorous acid compounds

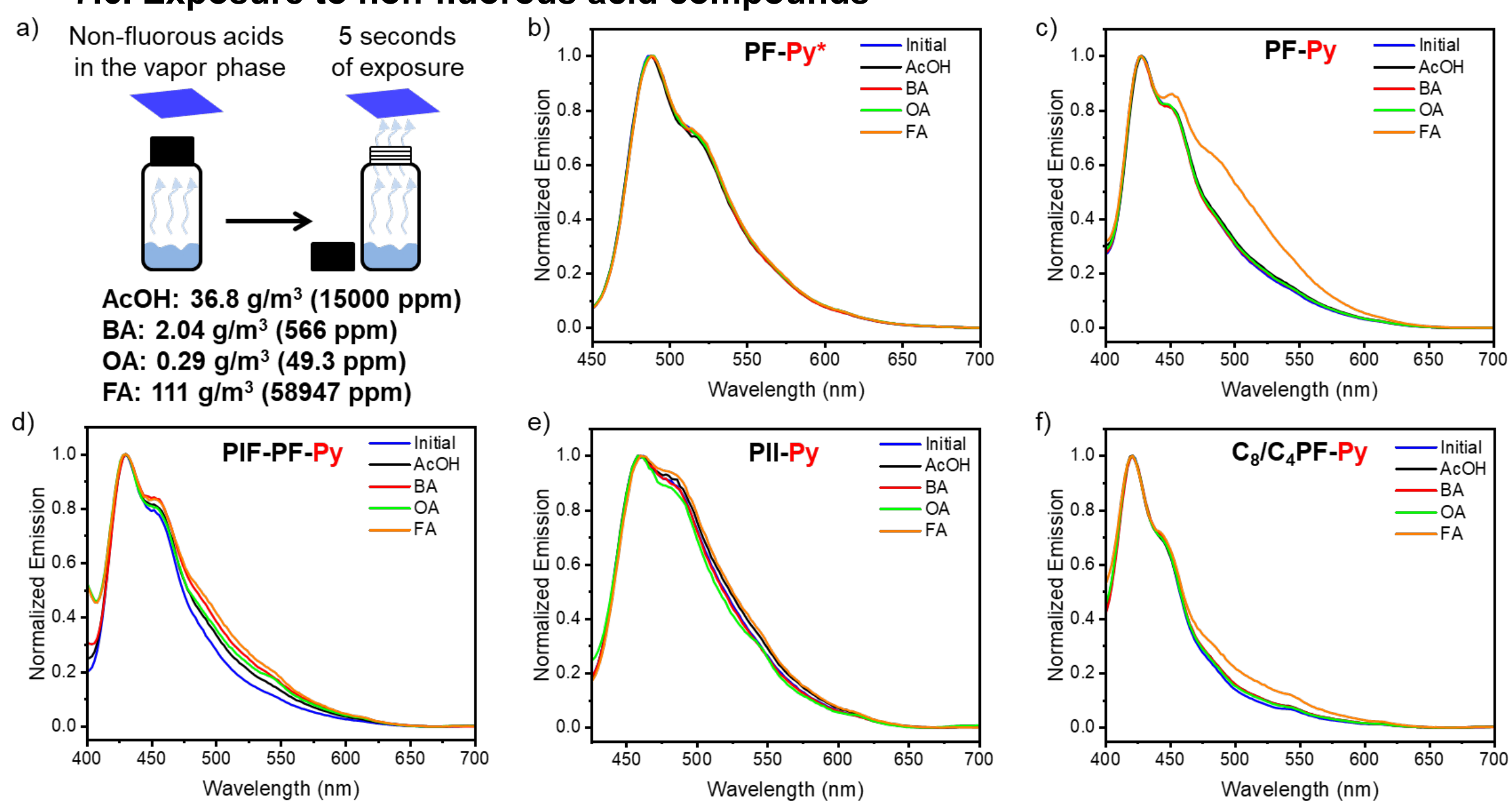

Figure S9. a) Graphical abstract of polymer thin films exposure to non-fluorous acid compounds in the vapor phase. The unactivated polymer emission is shown in blue. Black, red, green, and orange spectra represent fluorescence change in 15 seconds after a 5 second exposure to AcOH, BA, OA and FA, respectively. Polymers used: b) **PF-Py***, c) **PF-Py**, d) **PIF-PF-Py**, e) **PII-Py**, and f) **C_8/C_4PF-Py**.

7.7. Calibration curve of PFBS and PFOA

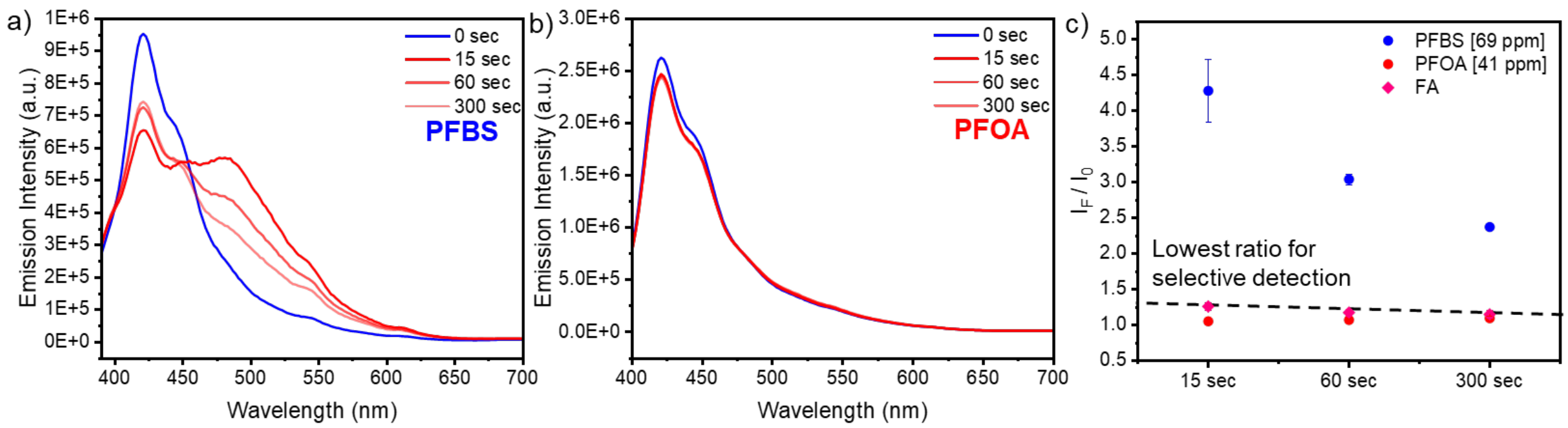

Figure S10. Variation of fluorescence intensity of **C8/C4PF-Py** thin films over time after 5 minutes of exposure to: a) PFBS, b) PFOA, c) Ratiometric fluorescence signals as a function of time after PFBS and PFOA exposure.

7.8. Film thickness effect during PFAS acids vapor detection

The thickness of thin films was measured in triplicate using independently prepared samples under different spin-coating conditions. They were measured by contact profilometry (Dektak 6M profilometer, Dektak32 analysis software).

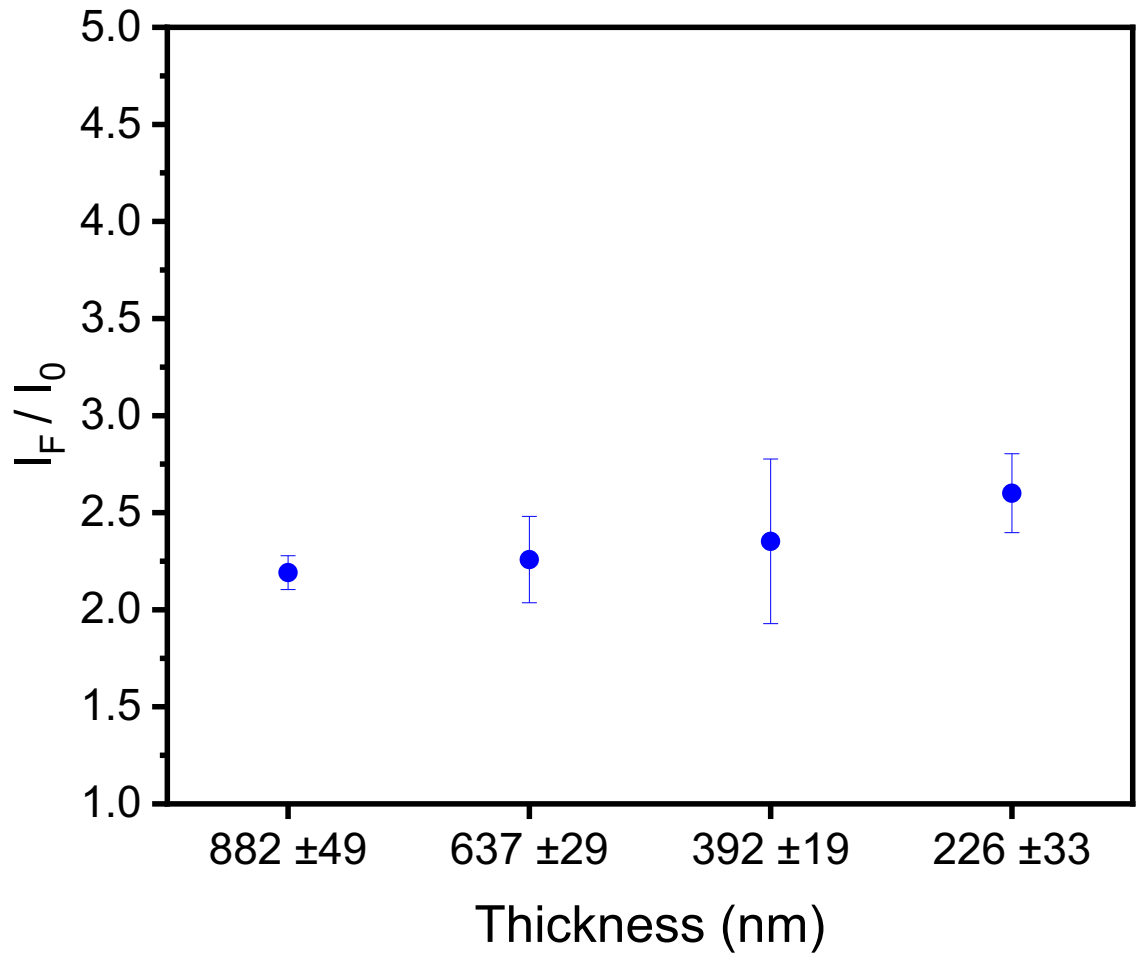

Figure S11. Ratiometric fluorescence signal of **C8/C4PF-Py** thin films with different thickness.

8. NMR spectra

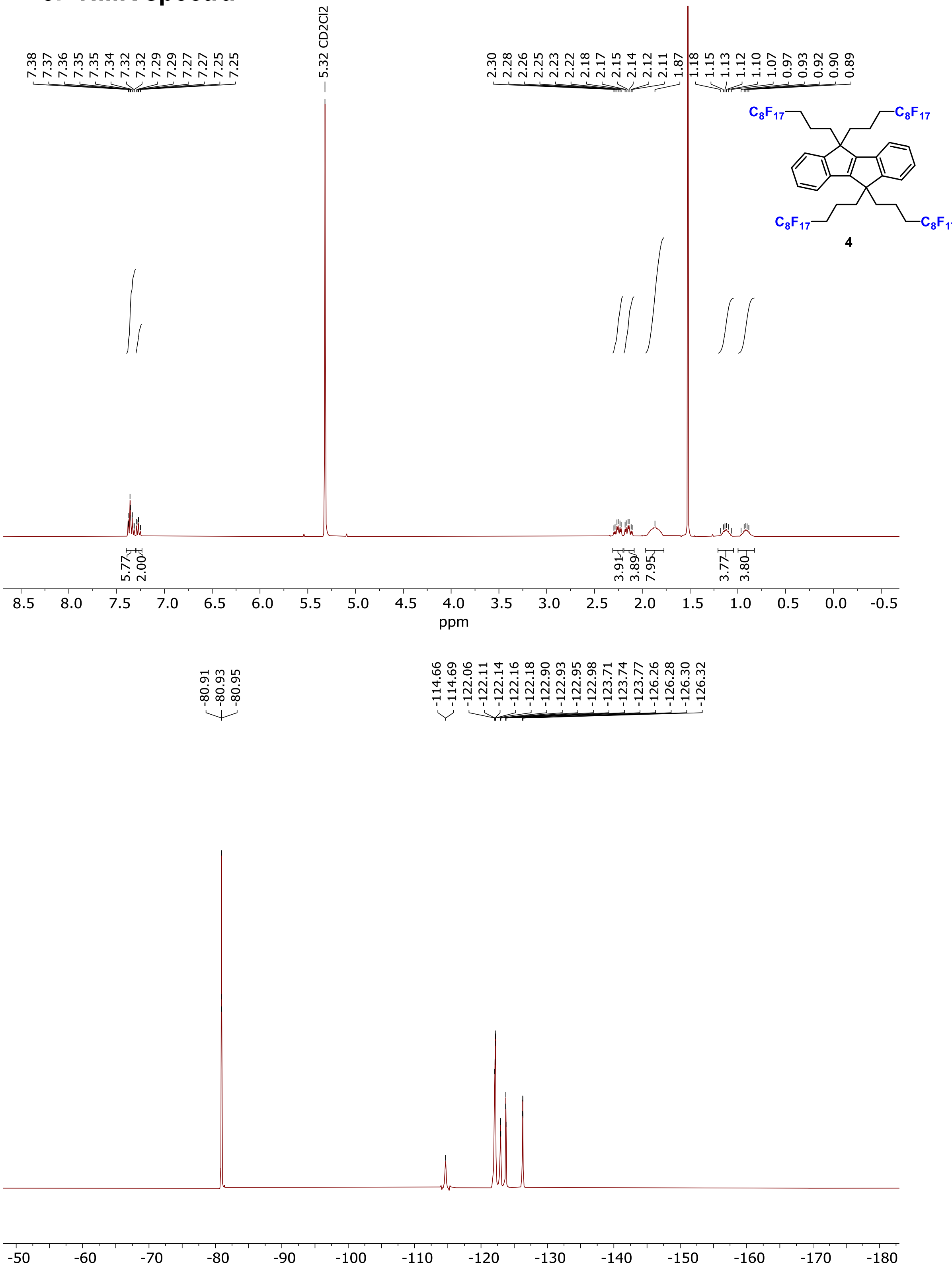

Figure S12. ^{1}H and ^{19}F spectra of compound **4**.

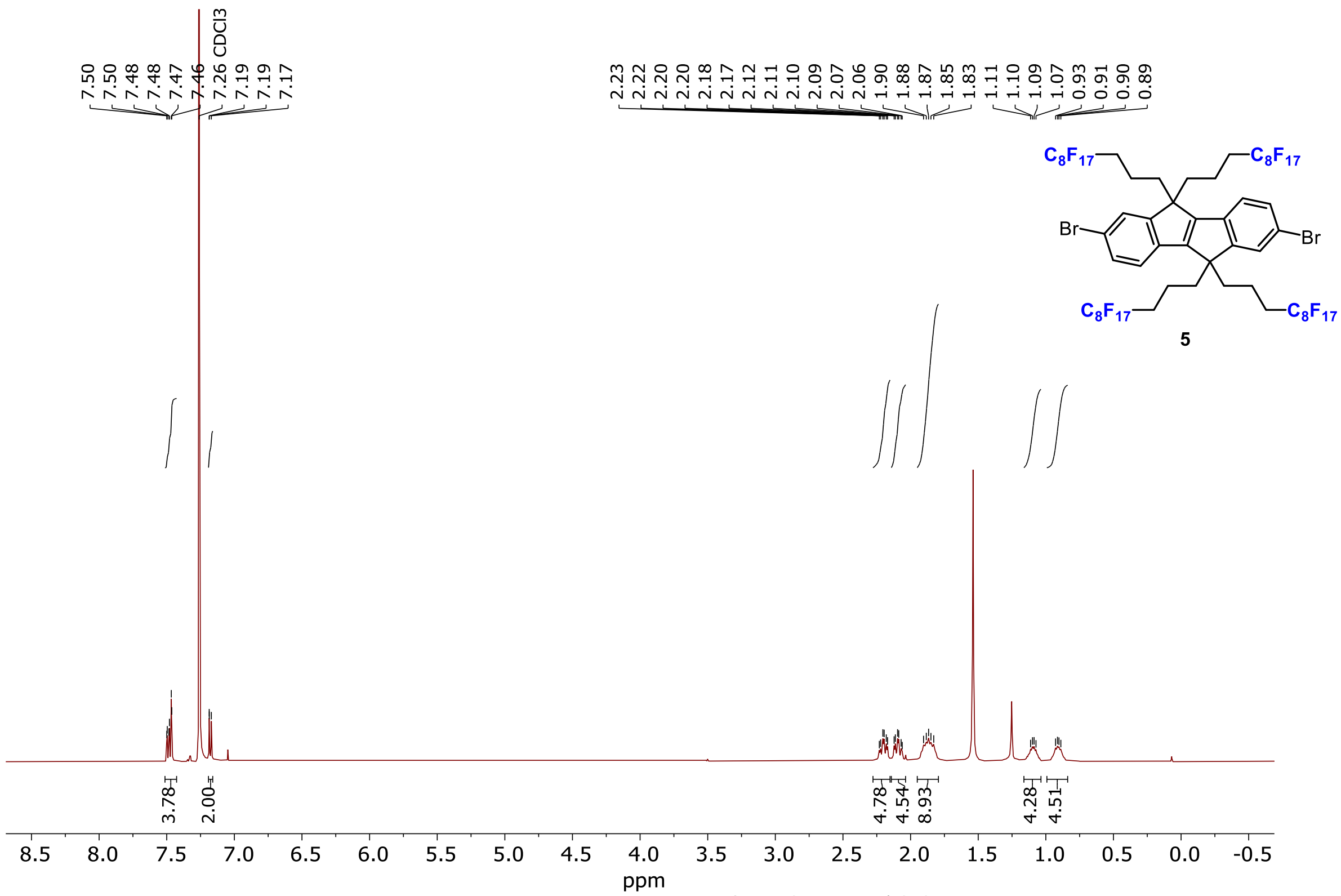

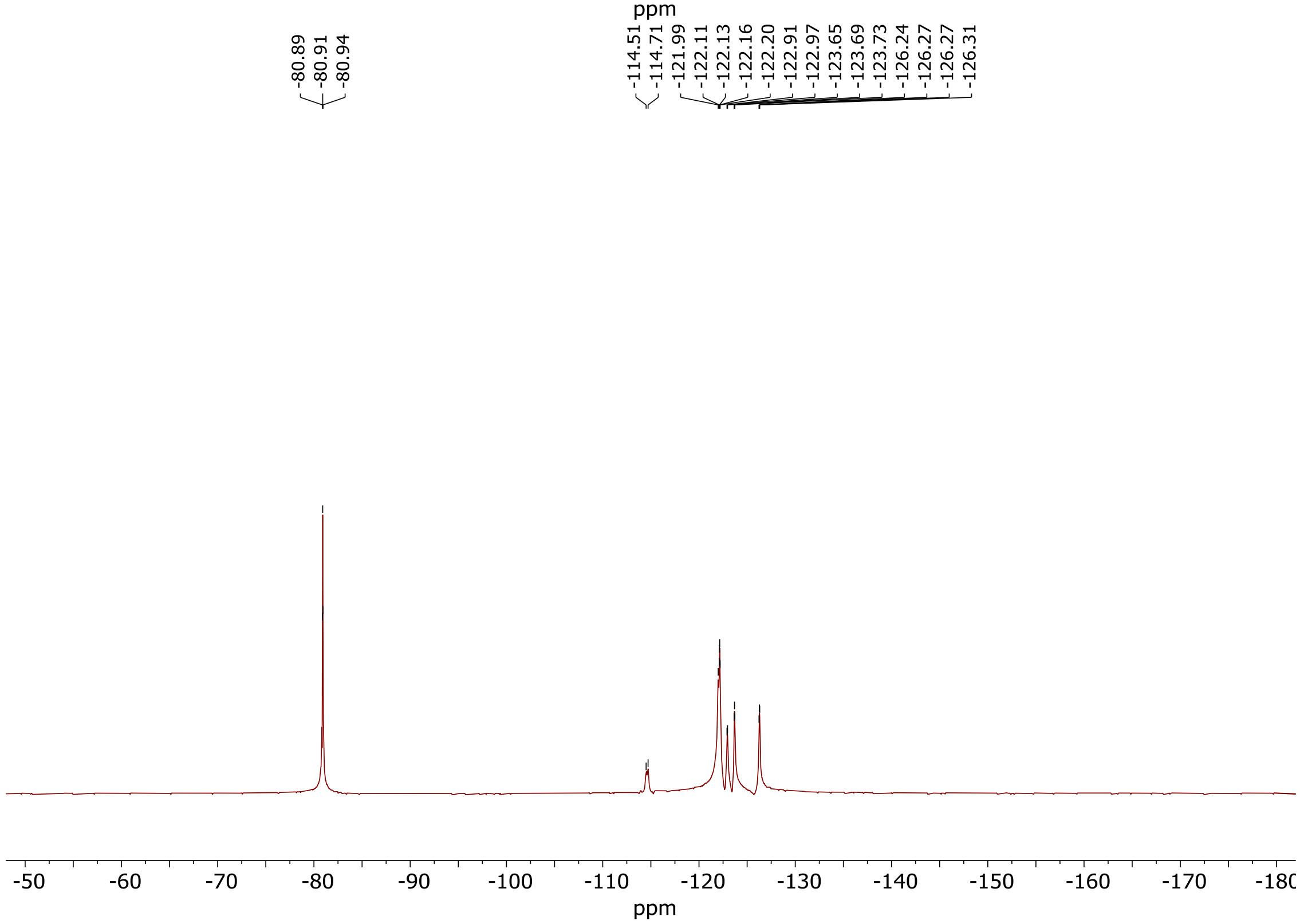

Figure S13. ^{1}H and ^{19}F spectra of compound **5**.

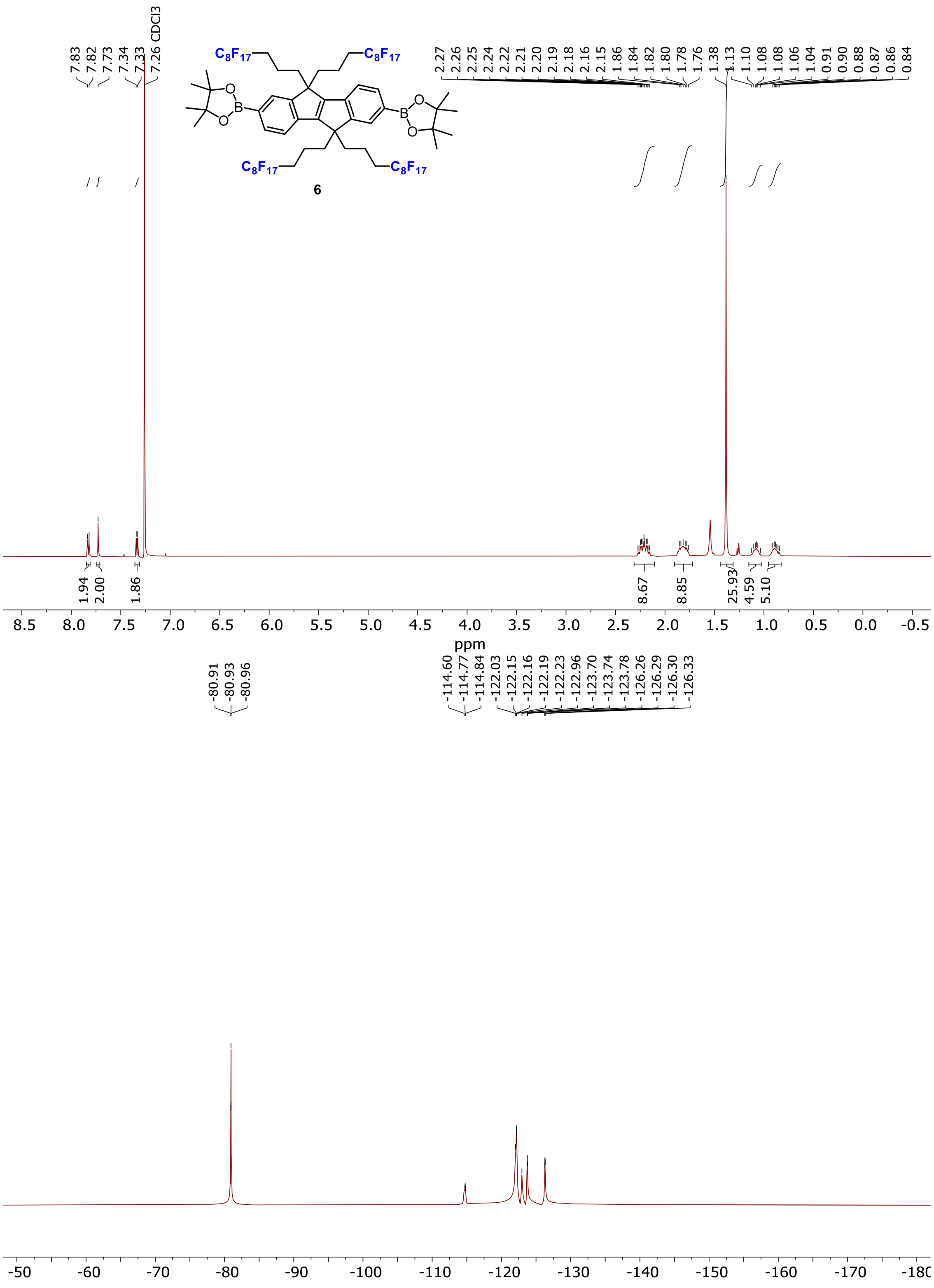

Figure S14. ^{1}H and ^{19}F spectra of compound **6**.

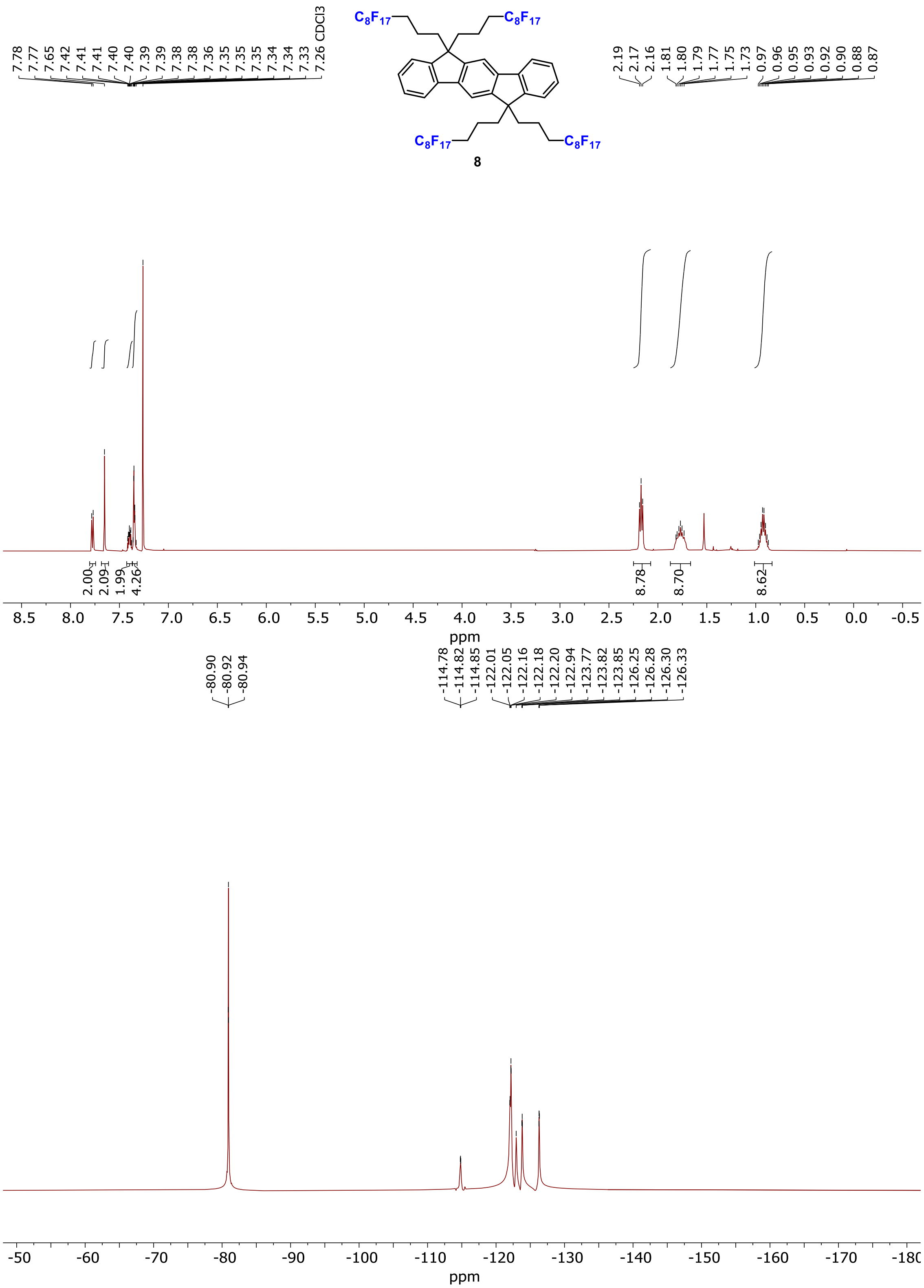

Figure S15. ^{1}H and ^{19}F spectra of compound **8**.

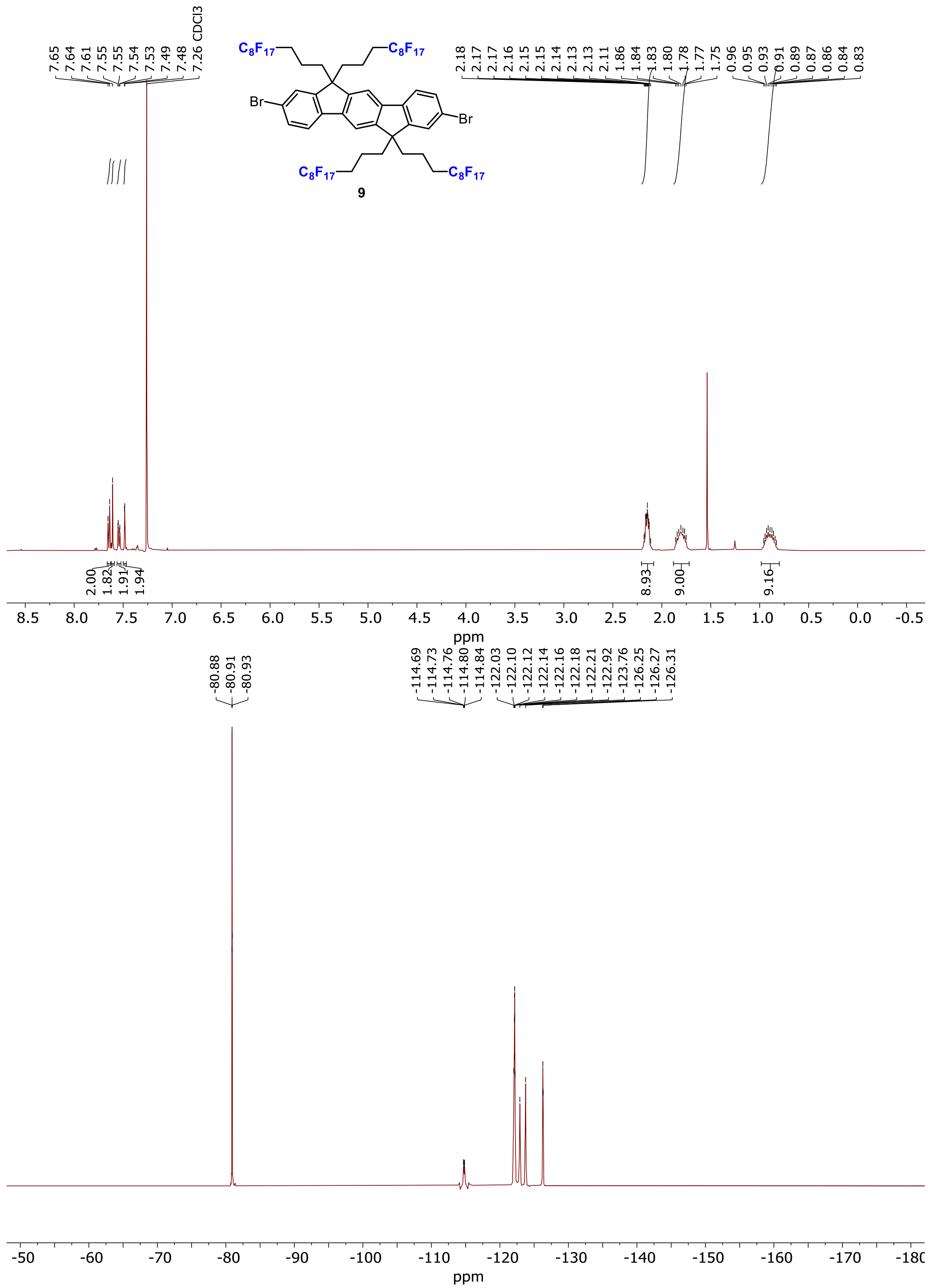

Figure S16. ^{1}H and ^{19}F spectra of compound **9**.

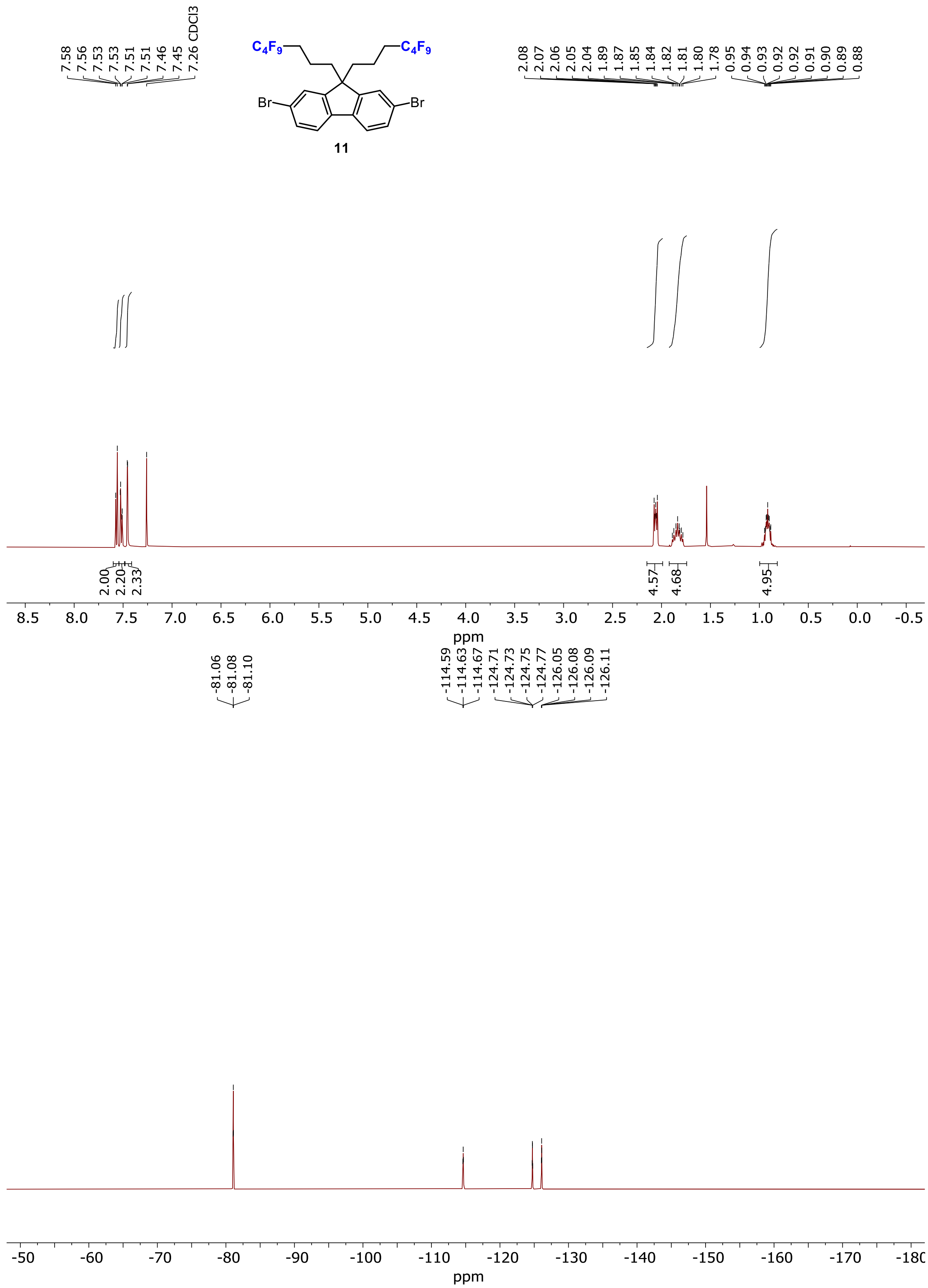

Figure S17. ^{1}H and ^{19}F spectra of compound **11**.

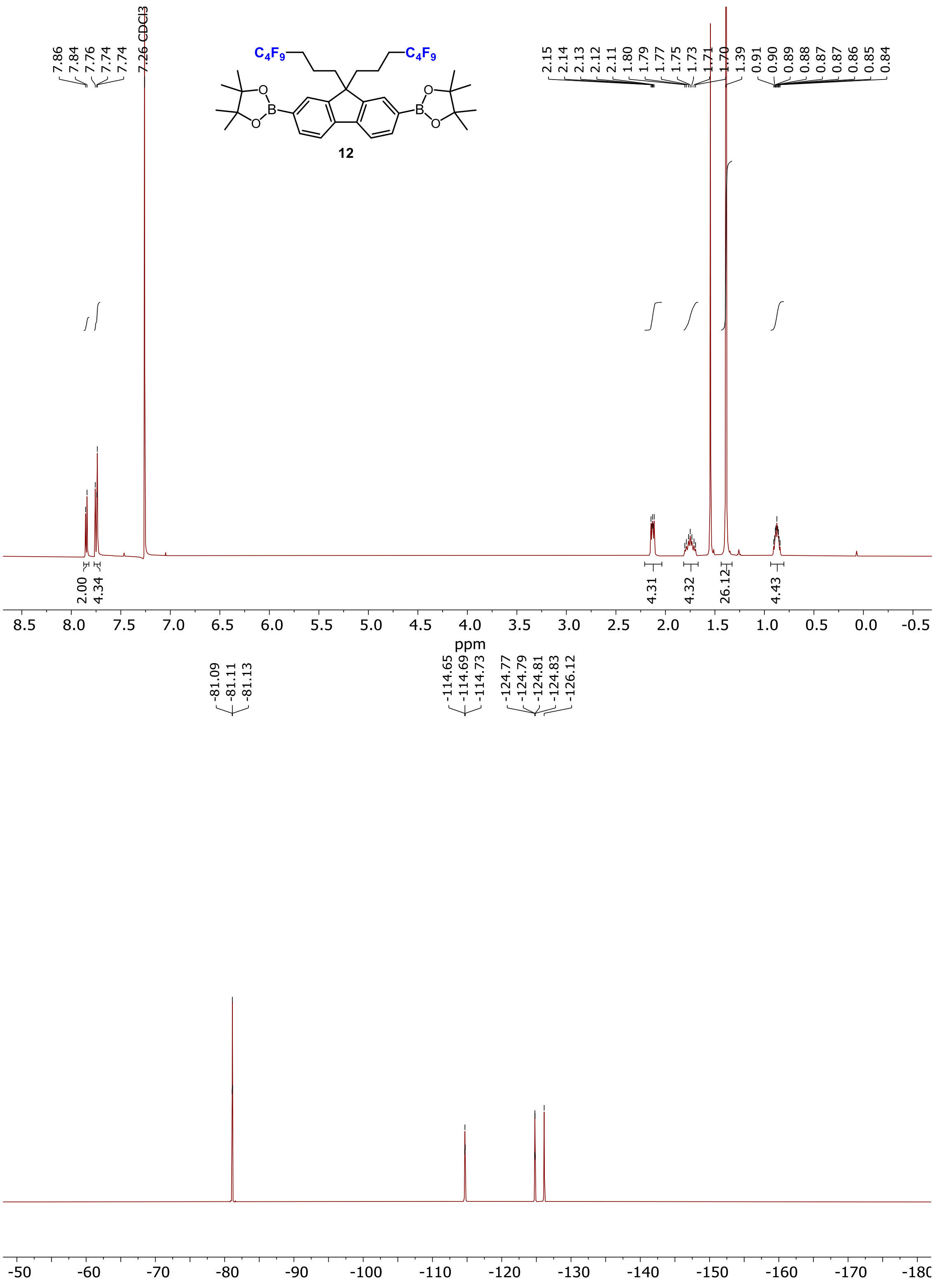

Figure S18. ^{1}H and ^{19}F spectra of compound **12**.

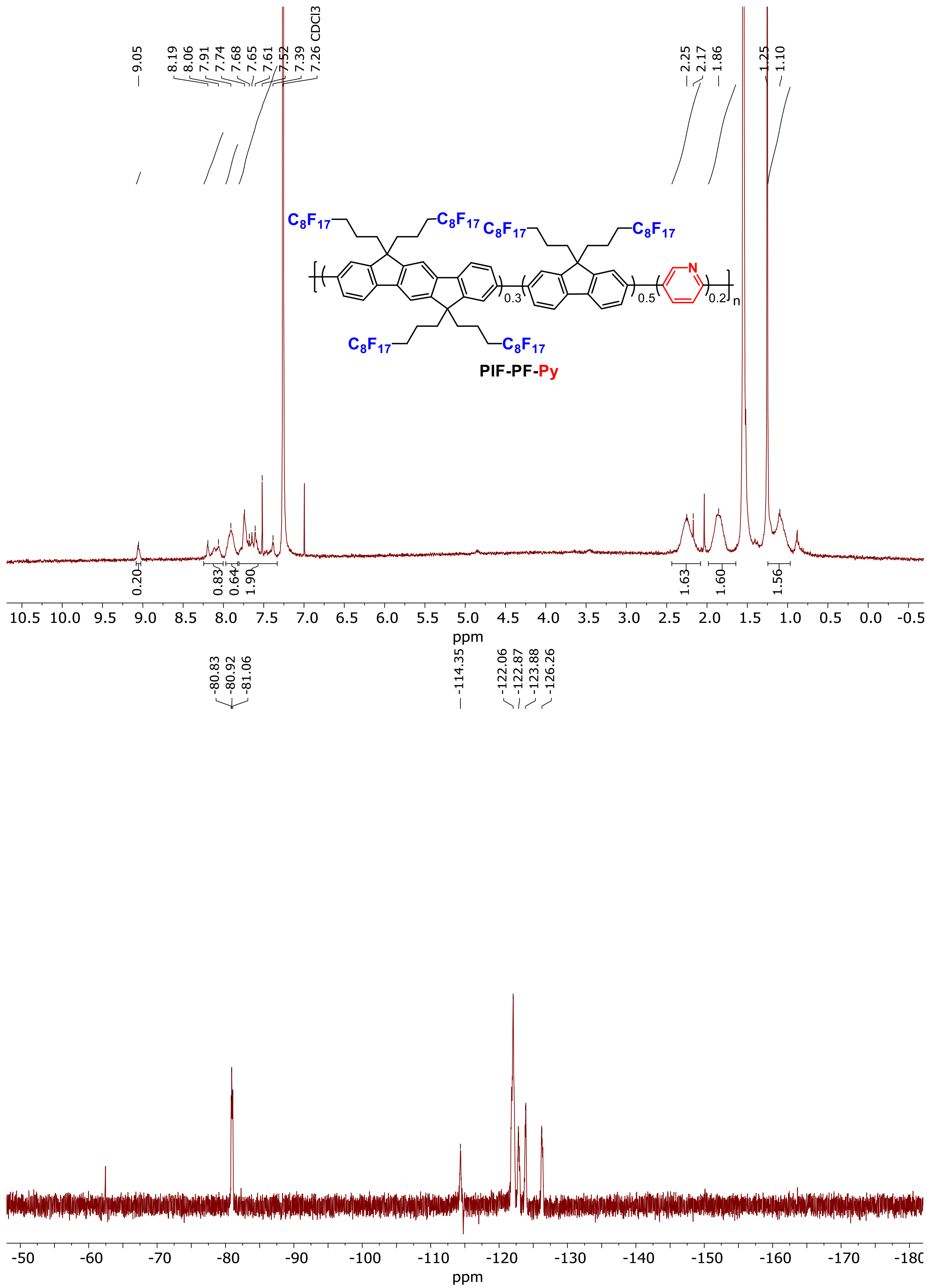

Figure S19. ^{1}H and ^{19}F spectra of polymer **PIF-PF-Py**.

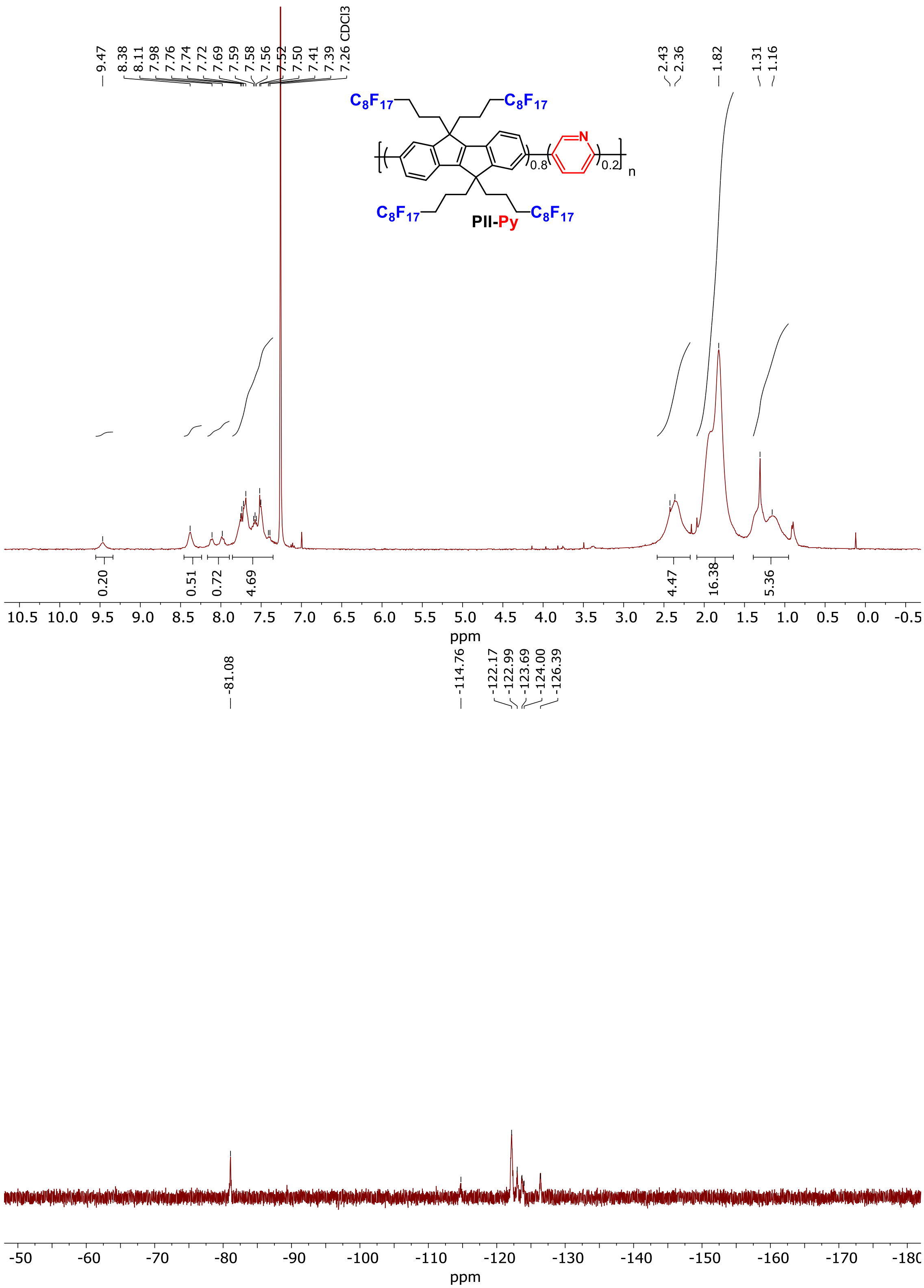

Figure S20. ^1H and ^{19}F spectra of polymer **PII-Py**.

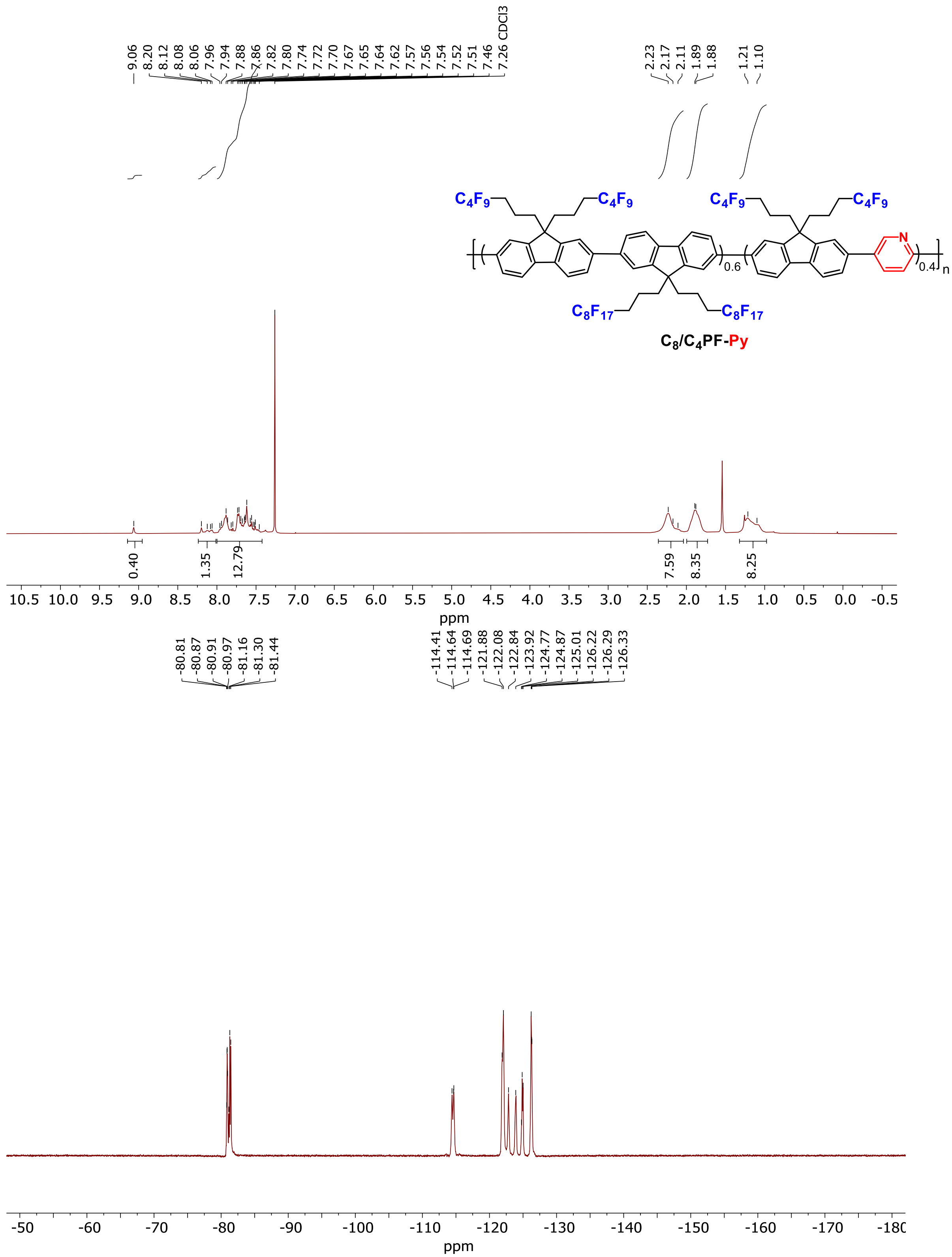

Figure S21. ^{1}H and ^{19}F spectra of polymer **C_8/C_4PF-Py**.

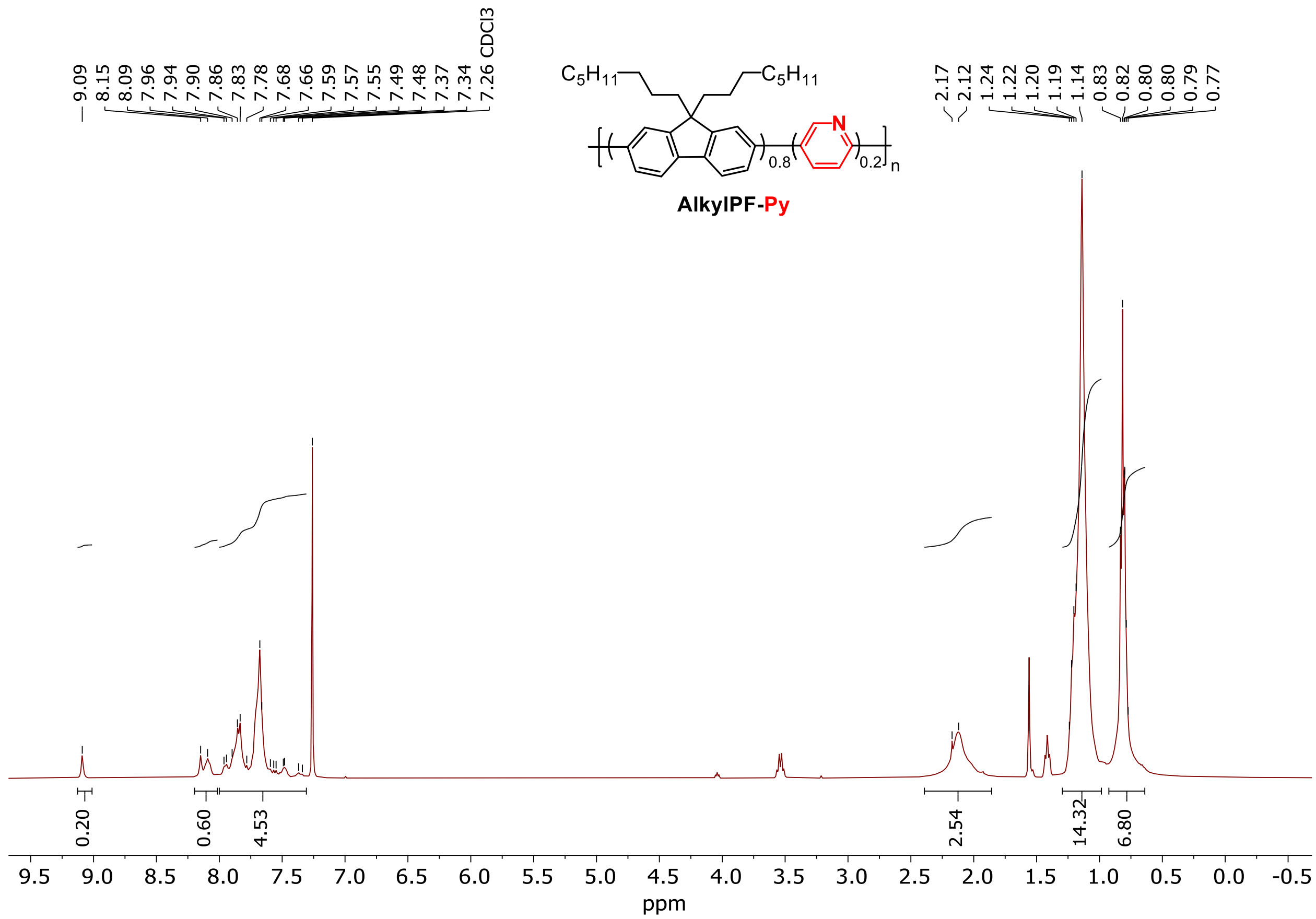

Figure S22. ^{1}H spectrum of polymer **AlkylPF-Py**.
